# Phase I-IIa clinical trial to evaluate the safety, feasibility and efficacy of the use of a palate mucosa generated by tissue engineering for the treatment of children with cleft palate: the BIOCLEFT study protocol

**DOI:** 10.1136/bmjopen-2024-093491

**Published:** 2024-12-05

**Authors:** Antonio España-López, Ricardo Fernández-Valadés, Elisa Cubiles, Ingrid Garzón, Miguel Angel Martin-Piedra, Víctor Carriel, Fernando Campos, Adoración Martínez-Plaza, Daniel Vallejo, Esther Liceras-Liceras, Jesús Chato-Astrain, Oscar Dario García-García, David Sánchez-Porras, Paula Ávila-Fernández, Miguel Etayo-Escanilla, Blanca Quijano, Elisabet Aguilar, Antonio Campos, Gloria Carmona, Miguel Alaminos

**Affiliations:** 1 Craniofacial Malformations and Cleft Lip and Palate Management Unit (Unidad de Fisurados Labiopalatinos y Malformaciones Craneofaciales), University Hospital Virgen de las Nieves, Granada, Spain; 2 Department of Stomatology, Faculty of Dentistry, University of Granada, Granada, Spain; 3 Instituto de Investigación Biosanitaria ibs.GRANADA, Granada, Spain; 4 Division of Pediatric Surgery, University Hospital Virgen de las Nieves, Granada, Spain; 5 Tissue Engineering Group, Department of Histology, Faculty of Medicine, University of Granada, Granada, Spain; 6 Fundación para la Investigación Biosanitaria de Andalucía Oriental (FIBAO), Granada, Spain; 7 Andalusian Network for the Design and Translation of Advanced Therapies (And&tAT/RAdytTA) - Fundación Andaluza Progreso y Salud, MP. Junta de Andalucía, Seville, Spain; 8 Division of Oral and Maxillofacial Surgery, University Hospital Virgen de las Nieves, Granada, Spain

**Keywords:** Cleft Palate, Clinical Trial, Cleft Lip, Histology

## Abstract

**Introduction:**

The current gold standard treatment for patients with orofacial clefts is surgical repair of the palatal defect (uranostaphylorrhaphy), which is associated with growth defects and hypoplasia of the maxillofacial structures. This trial aims to evaluate the potential of a bioengineered artificial palate mucosa, created through tissue engineering with autologous stromal and epithelial cells and nanostructured fibrin–agarose biomaterials, to enhance treatment outcomes for patients with unilateral cleft lip and palate.

**Methods and analysis:**

This phase I-IIa clinical trial aims to evaluate the feasibility and biosafety of a procedure involving grafting bioartificial palate mucosa onto the areas of denudated bone in patients undergoing uranostaphylorrhaphy. The control patients will undergo standard surgical treatment. Five patients will be included in the first biosafety phase. In the second phase, 10 patients will be randomly assigned to the intervention or control group (1:1). The intervention group will undergo standard surgical treatment followed by the application of autologous bioartificial palate mucosa. Feasibility will be analysed at the time of surgery. Nine postimplant visits will be scheduled over a 2-year follow-up period, in which local and systemic biosafety will be investigated by determining graft evolution, including signs of necrosis, rejection, inflammation and patient factors. Preliminary signs of efficiency will be explored by sequentially evaluating craniomaxillofacial development, hearing impairment, speech capability and quality of life of the family. The research will be published in journals and posted in the relevant repositories when available.

**Ethics and dissemination:**

This study has been approved by the Committee of Ethics in Research with Medicinal Products (CEIm) and authorised by the Spanish Medicines Agency (AEMPS). The results of this study will be published in peer-reviewed journals.

**Trial registration number:**

NCT06408337; ClinicalTrials.gov: EuclinicalTrials. eu: 2023-506913-23-00.

**STRENGTHS AND LIMITATIONS OF THIS STUDY**

- This study describes the protocol of an advanced therapies clinical trial approved by the Spanish Medicines Agency.
- The clinical trial will assess the feasibility and biosafety of a tissue-engineered, bioartificial palate mucosa for treating children born with orofacial cleft.
- The single-centre design could limit the extrapolation of the results.
- The sample size of 15 patients is small, although it could be sufficient for an initial feasibility and biosafety analysis.

## Introduction

 Orofacial cleft (OFC) is a congenital defect that affects 1:700 to 1:1000 live births and is the most common congenital defect in developed countries, after only Down syndrome.^1^ This condition may affect the patient’s lip, palate or both structures and is usually clinically detected at birth when the different maxillofacial processes are unfused, manifesting as a defect in the lip or hard palate and soft tissues.^2^ OFC is associated with serious physical, psychological and social impacts affecting patients and their families.^3^ Aside from the primary malformation, patients and their families must also contend with complex surgical interventions and interdisciplinary medical-surgical treatments.^4 5^ Typically, management is long and complex and includes surgical corrections, orthodontic treatments, bone grafts, rhinoplasty, psychotherapy and speech therapy.^4^ Moreover, the outcomes of the standard treatments are not always optimal.^6^

The gold standard surgical treatment for palatal repair is uranostaphylorrhaphy, which involves obtaining mucosal flaps from the remaining areas of the hard palate and suturing the flaps in the midline to generate a physical barrier between the oral and nasal cavities.^7^ Unfortunately, this procedure is associated with impaired maxillofacial growth and hypoplasia of the craniofacial structures. The denudation of the palatal bone disrupts facial growth and development.^8 9^ In contrast, other surgical techniques, such as Furlow palatoplasty, which is a buccal myomucosal flap procedure, reportedly offer improved results with fewer impacts on maxillary bone growth in certain patients.^10^ In patients with cleft lip and palate, the lip is routinely repaired (cheiloplasty) when the patient is 3–6 months old, whereas the palate is usually repaired surgically approximately 1 year later when the patient is 15–18 months old.^11^

Bioartificial tissues generated by tissue engineering (TE) have been proposed to improve outcomes following surgical repair of OFC. TE combines live cells with biocompatible biomaterials and growth factors to generate functional tissue substitutes to replace damaged tissues and organs.^12^ In OFC, only a few models of bioartificial tissues generated by TE have been described and evaluated in animal models.^13^ One such model is BIOCLEFT, a palatal mucosa substitute generated by our research group using nanostructured fibrin–agarose biomaterials combined with oral mucosal stromal and epithelial cells, including fibroblasts and keratinocytes. Nanostructured fibrin–agarose biomaterials have shown good biocompatibility and promising clinical results in patients with severe corneal ulcers^14^ and extensive skin burns.^15^ The application of this palate substitute in an animal model of palate damage in newborn rabbits resulted in significant improvement in the development and growth of hard and soft tissues, with a positive outcome in most animals.^16^ After a thorough evaluation and characterisation of the BIOCLEFT product at the biomechanical, histological, histochemical and immunohistochemical levels,^16^,^18^ we obtained approval (date: 20 November 2023) from the Spanish Medicines Agency (*Agencia Española de Medicamentos y Productos Sanitarios, AEMPS*) to generate BIOCLEFT as an advanced therapy medicinal product (ATMP) and to implement the BIOCLEFT clinical trial in patients affected by cleft palate.

Here, we present the protocol for the phase I-IIa BIOCLEFT clinical trial (protocol V.2, date of approval: 19 October 2023. This trial will determine the feasibility and biosafety of this novel TE product in children with cleft palate and will preliminarily evaluate treatment efficacy.

## Methods and analysis

### Study design

The BIOCLEFT clinical trial will be a phase I-IIa-controlled, open-label, randomised, unicentric advanced therapy trial to evaluate the feasibility and safety of an autologous palate mucosa substitute generated by TE in children with cleft palate. The trial coordinator is Dr Ricardo Fernández-Valadés and the project principal investigator is Dr Miguel Alaminos. The sponsor is the Andalusian Network for Design and Translation of Advanced Therapies and Foundation for Biomedical Research in Eastern Andalusia (FIBAO).

The control group will undergo the gold-standard surgical repair procedure, uranostaphylorrhaphy. In contrast, the study group will be treated with the same procedure, followed by the implantation of a BIOCLEFT substitute used to cover the lateral areas of denudated bone. The professionals involved in the trial will not be blinded to the study.

The uranostaphylorrhaphy procedure used in both groups of patients consists of the closure of the central cleft by suturing the edges of the cleft together. First, the soft tissue (palate mucosa) is carefully detached from the subjacent palate bone on both sides of the palatal defect, taking care not to damage the vascular supply of the soft tissue. The right and left tissues are drawn toward the midline defect and sutured together using silk stitches. This procedure allows a physical barrier to form between the oral and nasal cavities using the palate mucosa; however, the palate bone is left denudated on both sides of the palate grafts. Finally, the uvula, soft palate and faringopalatine muscles are repaired and sutured to re-establish the normal anatomy of the human pharynx structures.

The first five patients will be sequentially recruited in the initial phase of the clinical trial. As an early phase clinical trial, a safety period of 30 days was established between patients to ensure that the previous patient did not experience any adverse effects due to the implant before subjecting the next patient to the implant. This safety period is a common requirement of national medicine agencies for novel products whose biosafety levels have yet to be determined.^19^ The Independent Data Security and Monitoring Committee will perform an interim biosafety analysis after the last patient included in the initial phase reaches 1.5 months of follow-up. This committee consists of five members that are independent from the sponsor and are free from any competing interests. If the safety analysis reports that the product is safe, the trial will continue to the second phase. Otherwise, the clinical trial will be terminated.

**Figure 1:**
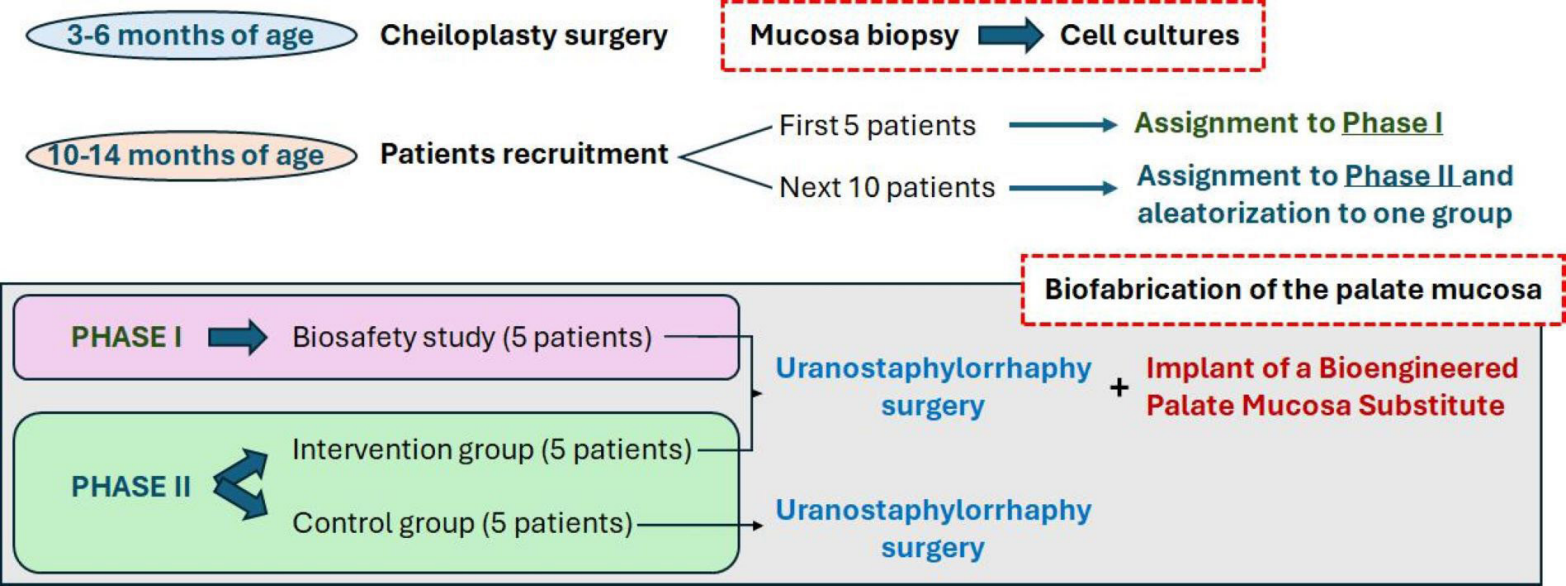
Design of the BIOCLEFT clinical trial. The different phases and groups of patients are represented at each stage of the trial.

In the second phase, 10 additional patients with cleft palate will be recruited and randomly assigned to one of the following groups (figure 1):

1. Control group (n=5), these patients will receive the gold-standard uranostaphylorrhaphy treatment without applying any grafting material.
2. Intervention group (n=5). These children will receive the gold-standard treatment, followed by implantation of the BIOCLEFT artificial palate mucosa, as is the case for patients in the initial phase of the trial. Consequently, this group’s total number of patients will be 10 (five in the initial phase and five in the second phase).

This clinical trial was initiated on 17 April 2024. At the time of submission of the present protocol, the clinical trial was in the phase of recruiting five patients for the initial phase of the trial. The study is expected to be completed by 17 December 2028.

### Patients and inclusion and exclusion criteria

Children with cleft palate, specifically unilateral cleft lip, will be included in this study. Patients will be recruited at the age of 10–14 months following the cheiloplasty procedure. As described in the Surgical Procedure section, all patients with non-syndromic cleft palate and unilateral cleft lip will donate a small oral mucosa sample during cheiloplasty. This sample corresponds to the tissue that is usually discarded after cheiloplasty.

Inclusion criteria:

- Paediatric patients, of both genders.
- Diagnosis of total unilateral non-syndromic cleft lip and palate that will undergo surgery for correction.
- Children who have previously donated a sample of oral mucosa during the cleft lip repair procedure (cheiloplasty).
- Informed consent signed by one or both parents (or legal guardian) adequately informed of the study and willing to follow the trial procedures and instructions.

Exclusion criteria:

- Active infectious diseases.
- Allergies or hypersensitivity to any of the components or excipients of the investigational product.
- Severe haematological disorders/blood dyscrasias.
- Severe hepatic or renal dysfunction/failure.
- Serious endocrine disorders/dysfunctions.
- Malignant neoplasms.
- Active HIV, hepatitis B virus (HBV) or hepatitis C virus (HCV) infection.
- Metabolic bone diseases (Paget’s disease, hypercalcemia, etc).
- Children with cleft lip and palate who present other congenital malformations that, in the researcher’s opinion, could affect the outcomes of the trial or the interpretation of results.
- In the opinion of the investigator, any other pathologies that should not be included in the trial for medical or social reasons.

### Surgical procedure

All patients enrolled in the study will be treated at the Craniofacial Malformations and Cleft Lip and Palate Management Unit (CMU) of the University Hospital Virgen de las Nieves of Granada, Spain. According to the unit’s management protocols, all patients will first undergo cheiloplasty to repair the lip defect. During cheiloplasty, oral mucosal biopsies will be obtained and transferred to a Good Manufacturing Practice facility located at the Advanced Therapies Platform of the ibs.GRANADA Research Institute and the University Hospital Virgen de las Nieves of Granada, coordinated by the Andalusian Network for the Design and Translation of Advanced Therapies. From these biopsies, epithelial cells (keratinocytes) and stromal cells (fibroblasts) will be isolated using enzymatic digestion methods, cultured and expanded as described previously.^16 18^ The cell cultures will be cryopreserved for delayed use.

The palatal defect will be repaired in all patients by applying a modified von Langenbeck uranostaphylorrhaphy surgical technique when the patient is approximately 14–18 months old. This procedure involves making two medial and lateral incisions at each side of the palatal cleft, followed by careful detachment of the soft tissue from the palatine bone without damaging its vascular pedicle, to generate a pediculated flap on each side of the palate.^20^ Both flaps are then sutured together in the midline of the palate defect to physically separate the oral and nasal cavities. To maintain velopharyngeal function, the muscles are also detached and repaired using sutures.^21^

For children in the intervention group, a BIOCLEFT palate mucosa substitute generated by TE will then be surgically applied to cover the denudated areas of the palatine bone on both sides of the hard palate that were exposed during the von Langenbeck surgery. BIOCLEFT will be generated as an ATMP at the Advanced Therapies Platform using autologous stromal and epithelial cells previously cultured from the biopsies taken during cheiloplasty. First, a stromal layer is fabricated using fibrin–agarose biomaterials with cultured fibroblasts. In brief, per mL of volume, 760 µL of human plasma obtained from plasma donors will be combined with 15 µL of tranexamic acid (Amchafibrin 5 mg/mL, Rotapharm, Monza, Italy), 100 µL of 2% agarose melted in phosphate-buffered solution (Merck, Darmstadt, Germany), 50 µL of CaCl_2_ at a concentration of 1% (Braun, Kronberg, Germany) and 75 µL of Dulbecco’s modified eagle medium (Merck).^22^ The patient’s keratinocytes (1 00 000 cells/mL of stromal substitute) will be subcultured on top of the stromal substitute to generate an epithelial layer. Palate mucosa substitutes are generated on porous culture inserts to promote epithelial stratification and differentiation using the air–liquid culture technique as previously described.^23 24^ Finally, the substitutes are subjected to plastic compression nanostructures to improve the biomechanical properties of the product, as described in the previous studies.^17^ This palate mucosa substitute will be applied to the denudated palatine bone on both sides of the palate defect and fixed using resorbable suture material.

### Outcomes, measures and variables

**Figure 2:**
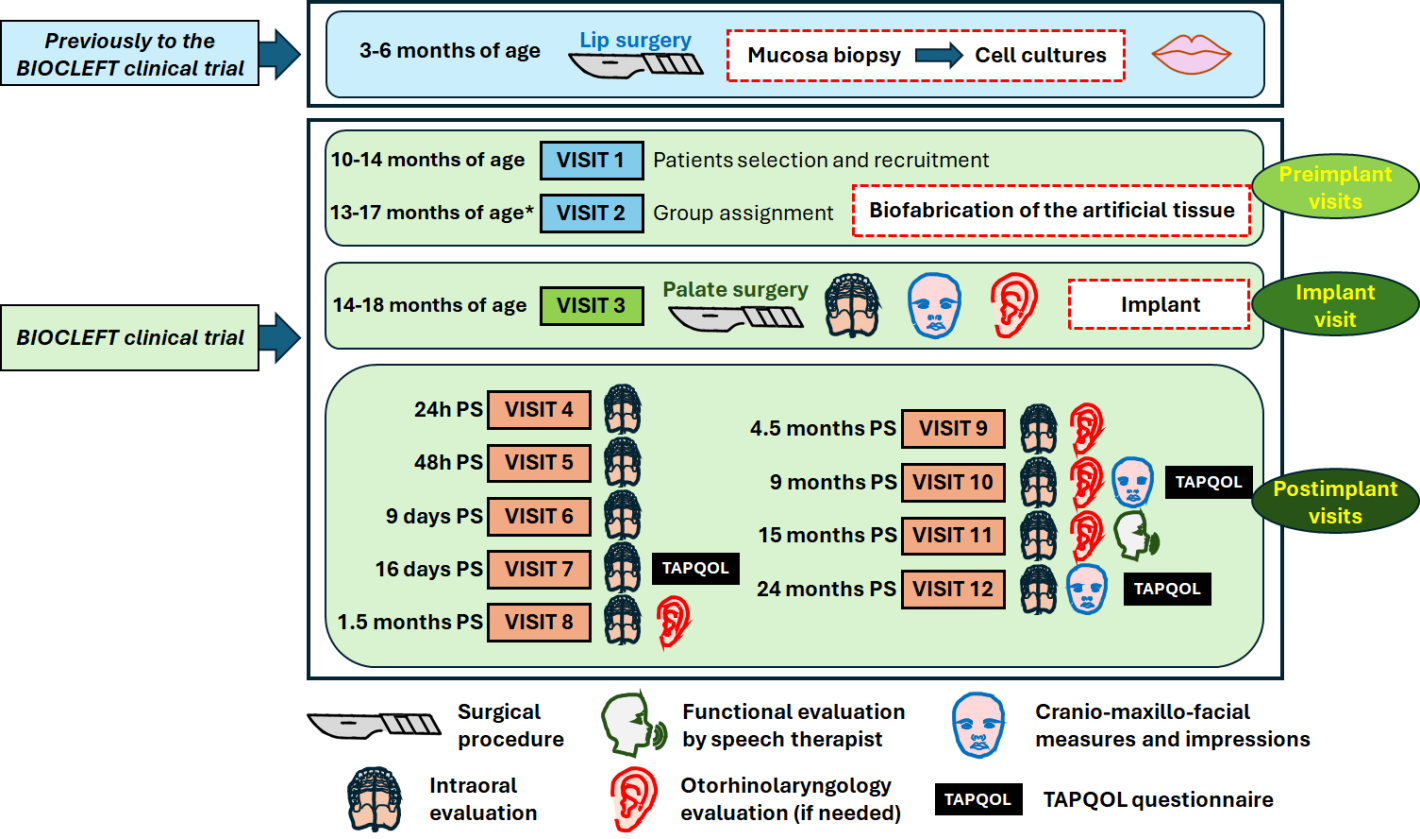
Stages of the BIOCLEFT clinical trial and investigations that will be conducted at each visit. The approximate moment when each visit will be programmed is shown to the left of the visit number, and the relevant analyses are represented with symbols (explained below the figure). PS, postsurgery; TAPQOL, TNO-AZL Preschool Quality of Life. *Visit 2 will take place approximately 30 days before the surgical procedure.

The clinical trial was designed with two preimplant visits (visits 1 and 2), one visit at the time of surgical repair of the palatal defect (visit 3), and nine postimplant evaluation visits (visits 4–12), with the last visit 24 months after uranostaphylorrhaphy (figure 2).

- Preimplant visits: the first visit will occur when the child is approximately 12 months old (range, 10–14 months) according to the current follow-up and treatment protocols applied at the CMU. Patients will be evaluated at this visit, and patients satisfying all the inclusion criteria will be selected and recruited for the trial. Informed consent will be obtained from all the parents or caregivers of the patients. The second visit will confirm the patient’s suitability for recruitment, and each patient will be randomly assigned to either the control or intervention group. The first five patients who meet the inclusion criteria will be assigned to phase I of the trial. The second visit will occur when the patient is 13–17 months old, approximately 30 days before uranostaphylorrhaphy.
- Implant visit (14–18 months of age): at the time of uranostaphylorrhaphy, with or without implantation of BIOCLEFT), the patient will be evaluated under general anaesthesia. Before uranostaphylorrhaphy, the patient’s palate cleft will be measured. The patient’s head, face, mouth and palate (craniomaxillofacial images) will be photographed, and palatal impressions in alginate gels will be obtained. These impressions will be used to generate 3D models of the patient’s defects before surgical treatment. In addition, participants will be evaluated by a paediatric otorhinolaryngologist, and tympanostomy ventilation tubes will be implanted if necessary.
- Postimplant visits. The first two postimplant visits will occur 24 hours and 48 hours after uranostaphylorrhaphy when the patient is still at the hospital. During these visits, the patient’s general condition and the surgical area of the palate will be evaluated. Specifically, the placement of the implant will be assessed, and short-term side effects and complications will be monitored, including graft detachment, bleeding, necrosis, infection and other unexpected findings. Patients are routinely discharged 48 hours after surgery. The same parameters will be evaluated during the rest of the visits (visits 6 to 12), which will occur at 9 and 16 days and at 1.5, 4.5, 9, 15 and 24 months after discharge. The TNO-AZL Preschool Quality of Life (TAPQOL) questionnaire will be used to evaluate the patient’s family at visits 7, 10 and 12, and a functional evaluation of the child’s speech will be performed by a paediatric speech therapist at visit 11. If necessary, a paediatric otorhinolaryngologist will evaluate the patient at visits 8, 9, 10, 11 or 12.

As an initial clinical trial, the variables to be analysed are mainly related to the feasibility of the procedure and the biosafety of the implant. However, secondary variables related to initial efficacy will be preliminarily analysed, and the present trial will record some initial signs of implant efficiency. The following variables will be analysed:

1. Primary outcome measures.
  - Feasibility will be assessed using a questionnaire generated ad hoc for this trial that includes items related to the difficulties encountered during the grafting process, macroscopic aspects, consistency, handling and suturability of BIOCLEFT and other factors associated with the feasibility of the procedure (online supplemental material 1).
  - Biosafety will be determined by analysing the occurrence of serious and mild adverse or unexpected side events related to the implantation of BIOCLEFT, such as excessive bleeding, necrosis, infection, inflammation or other local or systemic reactions that could be related to the implant.
2. Secondary outcome measures.
  - Effects of the implants on surgical site healing. The regeneration and healing of the palatine bone defects generated during uranostaphylorrhaphy will be analysed by evaluating the surgical site at different time points.
  - Evaluation of aesthetic results. The appearance of the patient’s head and face will be assessed by analysing macroscopic photographs taken at different follow-up times. A specific aesthetic appearance assessment scale designed for children with OFC (online supplemental material 2) will be used. This scale was designed based on previous scales used in children with cleft palate.^25 26^
  - Preliminary evaluation of craniomaxillofacial growth and development. Although the follow-up time of the present clinical trial is only 24 months, initial preliminary signs of the effects of the implant on craniomaxillofacial growth and development will be assessed by quantifying relevant measures and distances in the photographs and 3D models obtained from the patient at different time points.
  - Hearing evaluation. A paediatric otorhinolaryngologist will evaluate patients to detect any signs of hearing impairment or otologic defects. Once the otorhinolaryngologist has performed the initial hearing evaluation (preimplant visits), the need for follow-up postimplant visits will be assessed depending on whether the patient has any pathology.
  - Analysis of quality of life. The patients’ families will be asked to fill out the TAPQOL questionnaire for children aged 1–5 years to determine if the treatment improved the quality of life of the patients’ families. This questionnaire is widely used to analyse children’s quality of life and contains several items evaluating 12 aspects of children’s life, including sleeping, appetite, lungs, stomach, skin, motor functioning, communication, social functioning, behavioural problems, anxiety, positive mood and liveliness.^27 28^
  - Functional evaluation by a speech therapist. An expert paediatric speech therapist will determine whether the treatment influenced any relevant speech parameters, such as the ability to pronounce vowels and consonants, nasal escape, palate mobility, swallowing and articulation of functional language. For this purpose, a 2–3 min conversational speech sample will be tape recorded for further analysis, followed by a single-word articulation test to assess specific sounds. These tests evaluate the patient’s ability to articulate speech and the resonance patterns associated with speech (hypernasality, resonance of specific vowels and consonants, detection of articulation errors).

After the trial, patients will be follow-up and treated in the CMU following the standard protocols established in this unit.

### Data sharing and diffusion plan

The results obtained from this trial will be posted in public databases and repositories as soon as possible as part of the study’s data management plan. The results will be published in a specialised journal. Personal data and those subject to special ethical or legal issues will be protected and not published. The data management plan is provided in online supplemental material 3.

## Discussion

Despite the social and healthcare relevance of OFC, current treatments are still based on surgical techniques originally described many decades ago.^29^ Although these techniques allow surgeons to generate a physical barrier between the oral and nasal cavities, the final outcomes of these patients remain suboptimal,^6^ and new treatments are needed. Among the most promising alternatives are advanced therapies using bioartificial tissues that offer the possibility of promoting tissue regeneration in patients with severe conditions.^30^

Artificial tissues generated as ATMPs using TE increasingly demonstrate their potential clinical usefulness in diverse, complex pathologies. Moreover, artificial tissues have been successfully and safely used to treat patients with severe diseases.^14 15 31 32^ However, the clinical translation of TE products is still limited, and little experience is available, especially for specific pathological conditions.^33^ In addition, the regulatory frameworks associated with clinical translation are very complex, making using these products in humans challenging.^34^

Following the successful clinical application of other human artificial tissues based on the same nanostructured fibrin–agarose biomaterials, we designed the present BIOCLEFT clinical trial.^14 15^ As one of the first advanced therapy clinical trials applied to patients with OFC, the present trial was designed according to the requirements of the AEMPS as a phase I-IIa trial. In general, novel products, especially ATMPs, must be initially evaluated for biosafety, either in early-phase clinical trials or in the framework of hospital exemption and compassionate use.^35 36^ For the BIOCLEFT clinical trial, we obtained authorisation from the national regulatory agency in Spain instead of applying for hospital exemptions, as was done for a previous model of artificial skin generated by the group.^35^ This will provide stronger scientific evidence of the effects of the artificial palate mucosa and enable future centralised marketing authorisation in Europe without the restrictive conditions associated with hospital exemption.^36^

As an early-phase clinical trial, the present study mainly focuses on establishing the feasibility of implanting the artificial palate mucosa and determining patient safety. Establishing feasibility is essential because the BIOCLEFT medical product is grafted onto the denudated areas of the palatine bone of patients affected by OFC during uranostaphylorrhaphy. Additionally, this is the first artificial tissue generated by TE containing two different cell populations (stromal and epithelial cells) generated as an ATMP and applied to patients with OFC. Therefore, this study primarily aims to demonstrate that the method and product are practically achievable. Although feasibility studies are common in cell therapy,^37^ few studies have described the feasibility of using human tissue generated by TW in clinical settings. Moreover, safety is among the most important requirements of new drugs and medicinal products,^38^ especially bioartificial tissues generated by TE.^39^

This trial will also preliminarily analyse other parameters related to the clinical utility of the implant. However, given the short follow-up period in the present clinical trial (24 months post-implant follow-up), the efficacy between the control and intervention groups is unlikely to differ significantly. These preliminary results could be used to design future clinical trials in more advanced phases focused on determining the clinical efficacy of the palate mucosa substitutes generated by TE.

Regarding the analysis, biosafety will be analysed by examining the patient’s surgical site and evaluating the patient’s general situation and clinical parameters. Based on our previous experiences with other bioartificial tissues generated by TE,^14 15^ these analyses should be able to detect any possible side effects and complications effectively, both in the short and long term. Notably, the nanostructured fibrin–agarose artificial tissues previously generated by our research group typically show complete in vivo biointegration after 1 to 3 months.^15 40^ To assess the preliminary efficacy of the bioengineered product, we will use a combination of methods, including analysing the growth and development of the craniomaxillofacial structures, assessing the hearing ability of patients using otorhinolaryngology evaluation, analysing speech ability and using an established normalised questionnaire to evaluate the quality of life of children and their families.^41^ This array of evaluation methods will provide researchers with a preliminary idea of the functional effects of the implant on the patient and will reveal the possible positive effects of the therapy, which should be confirmed by further analyses.

The BIOCLEFT clinical trial has several limitations. First, the study will be performed with a small number of participants, which is typically associated with lower statistical power.^42^ However, initial biosafety studies usually include a small sample size, especially in ATMP studies. Moreover, the follow-up period may not be sufficient to identify the beneficial effects of the implant on the patient’s craniomaxillofacial growth, development and aesthetic appearance. If the present study demonstrates that the implant is feasible and safe for the patient, future advanced clinical trials should be conducted using longer follow-up periods to allow the patient’s craniomaxillofacial structures to grow and develop.

## Ethics and dissemination

This study was approved by the Committee of Ethics in Research with Medicinal Products, reference FIB-BIO-2023–03 (date of approval, 21 November 2023). As an advanced therapy study, the BIOCLEFT clinical trial was authorised by the Spanish Medicines Agency (*Agencia Española de Medicamentos y Productos Sanitarios, AEMPS*), reference 2023-506913-23-00/ID:10008 (date of approval, 21 November 2023). AEMPS will audit the development of the trial. The study was performed in compliance with the Declaration of Helsinki and the principles of Good Clinical Practice. All legal guardians signed an informed consent form before study entry (online supplemental material 4). This clinical trial was registered at ClinicalTrials.gov. The results of the clinical trials will be published in a peer-reviewed journal.

## supplementary material

10.1136/bmjopen-2024-093491 online supplemental file 1

10.1136/bmjopen-2024-093491 online supplemental file 2

10.1136/bmjopen-2024-093491 online supplemental file 3

10.1136/bmjopen-2024-093491 online supplemental file 4
