# Supplementary material for: Phase I-IIa clinical trial to evaluate the safety, feasibility and efficacy of the use of a palate mucosa generated by tissue engineering for the treatment of children with cleft palate: the BIOCLEFT study protocol
Source: BMJ Open. 2024 Dec 5;14(12):e093491. doi: 10.1136/bmjopen-2024-093491 (PMC11624797; doi:10.1136/bmjopen-2024-093491)
Supplement: online supplemental file 1 [file bmjopen-14-12-s001.pdf]

Supplementary Table S1. Questionnaire used to assess feasibility of the procedure.

**SURGEON SATISFACTION QUESTIONNAIRE. Each question must be rated using a Likert-like scale ranging from 1 to 5**	**VERY BAD (1)**	**BAD (2)**	**AVERAGE (3)**	**GOOD (4)**	**EXCELLENT (5)**
**1. GENERAL APPEARANCE AND ASPECT OF THE BIOCLEFT AUTOLOGOUS PALATE MUCOSA GENERATED BY TISSUE ENGINEERING USING NANOSTRUCTURED FIBRIN-AGAROSE BIOMATERIALS GRAFTED IN CHILDREN WITH CLEFT LIP AND PALATE**					
1.1. The product has an adequate aspect, is homogeneous and is devoid of any apparent defects?					
1.2. The size of the product is adequate for use in patients affected by cleft lip and palate?					
1.3. The color of the product is compatible with a normal tissue?					
1.4. Is it easy to identify both tissue layers of the product (epithelium and connective tissue)?					
**2. HANDLING THE BIOCLEFT PRODUCT**					
2.1. Can this product be easily extracted from the recipient in which the tissue substitute has been delivered to the operating room?					
2.2. Can this product be easily handled using surgical forceps?					
2.3. Can this product be easily trimmed and adapted to the surgical site using a scalpel or other surgical instrument?					
2.4. Can this product be easily placed at the palate defect area?					
2.5. Can this product be easily sutured at the palate defect area?					
2.6. Has this product the capabiity to adhere to the palate defect area?					
**3. RESULTS OF THE IMPLANT**					
3.1. Has this product efficiently covered the palate defect area?					
3.2. Once grafted, is the aspect of the implant adequate?					
3.3. Once grafted, has the implant a homogeneous aspect, without bubbles and without any detectable defects?					
3.4. Overall, do you think the implant of the product has been feasible?					
3.5. In general, do you think the implant of the BIOCLEFT product has been easy?					
