# Supplementary material for: Phase I-IIa clinical trial to evaluate the safety, feasibility and efficacy of the use of a palate mucosa generated by tissue engineering for the treatment of children with cleft palate: the BIOCLEFT study protocol
Source: BMJ Open. 2024 Dec 5;14(12):e093491. doi: 10.1136/bmjopen-2024-093491 (PMC11624797; doi:10.1136/bmjopen-2024-093491)
Supplement: online supplemental file 2 [file bmjopen-14-12-s002.pdf]

Supplementary Table S2. Questionnaire used to assess the patient's aesthetic appearance.

**PATIENT AESTHETIC EVALUATION. Each question must be rated using a Likert-like scale ranging from 0 to 4**	**NO IMPROVEMENT (0)**	**25% IMPROVEMENT (1)**	**50% IMPROVEMENT (2)**	**75% IMPROVEMENT (3)**	**100% IMPROVEMENT (4)**
**1. LIP EVALUATION**					
1.1. Lip symmetry					
1.2. Shape of the philtrum					
1.3. Visibility of the scar					
1.4. Symmetry of the dry/wet line					
1.5. Lip fullness					
**2. NOSE EVALUATION**					
2.1. Symmetry of the nose tip					
2.2. Symmetry of the nostrils					
