# Supplementary material for: Phase I-IIa clinical trial to evaluate the safety, feasibility and efficacy of the use of a palate mucosa generated by tissue engineering for the treatment of children with cleft palate: the BIOCLEFT study protocol
Source: BMJ Open. 2024 Dec 5;14(12):e093491. doi: 10.1136/bmjopen-2024-093491 (PMC11624797; doi:10.1136/bmjopen-2024-093491)
Supplement: online supplemental file 3 [file bmjopen-14-12-s003.pdf]

DATA MANAGEMENT PLAN (DMP)

Clinical trial title: Phase I-IIa, Randomized, Controlled, Open-label, Single-center Clinical Trial to Evaluate Safety, Feasibility, and Efficacy of the Use of BIOCLEFT in the Treatment of Patients with Cleft Palate (BIOCLEFT clinical trial)

Protocol number: FIB-BIO-2023-03

Sponsor: Fundación para la investigación Biosanitaria de Andalucía Oriental-Alejandro Otero (FIBAO)

Creator: Miguel Alaminos

Principal Investigators: Miguel Alaminos and Ricardo Fernández-Valadés

Affiliation: University of Granada and University Hospital Virgen de las Nieves, Granada, Spain

Template: DCC Template

ORCID iD: 0000-0003-4876-2672 (MA) and 0000-0001-8087-4311 (RFV)

Project abstract:

Introduction. Current gold-standard treatment for patients with orofacial cleft is the surgical repair of the palatal defect (uranostaphylorrhaphy), which is associated to growth defects and hypoplasia of the maxillofacial structures. This study will analyze the potential of a bioengineered model of artificial palate mucosa generated by tissue engineering using autologous stromal and epithelial cells and nanostructured fibrin-agarose biomaterials to improve the results of the treatment of patients with unilateral cleft palate and lip.

Methods and analysis. A phase I-IIa clinical trial was implemented to determine the feasibility and biosafety of a procedure in which a bioartificial palate mucosa is grafted on the areas of denudated bone in patients subjected to uranostaphylorrhaphy. Control patients will receive the standard surgical treatment. 5 patients will be included in the first biosafety phase of the study. A second phase will be implemented with 10 patients randomly assigned to the intervention or control groups (1:1). The intervention group will receive standard surgical treatment followed by application of an autologous bioartificial palate mucosa. Feasibility will be analyzed at the moment of surgery. 9 postimplant visits are scheduled in a 2-year follow-up period, in which local and systemic biosafety will be analyzed by determining the evolution of the graft (signs of necrosis, rejection, inflammation, etc.) and the patient. Preliminary signs of efficiency will be also explored by sequentially evaluating cranio-maxillo-facial development, hearing impairment, speech capability and the quality of life of the family. When available, results will be published in journals and posted in relevant repositories.

Ethics and dissemination. The study was approved by the Committee of Ethics in Research with Medicinal Products (CEIm) and authorized by the Spanish Medicines Agency (AEMPS). Results of the study will be published in peer-reviewed journals.

Trial registration. ClinicalTrials.gov: NCT06408337; Euclinicaltrials.eu: 2023-506913-23-00.

ID: 162342

Start date: 17-04-2024

Last modified: 27-10-2024

Grant number / URL: ICI19/00024

BIOCLEFT CLINICAL TRIAL

DATA COLLECTION

The data to be collected are described in the study protocol, and include:

- Participant information: ID, demographics, previous medical history.
- Inclusion/exclusion criteria.
- Treatment details.
- Outcome Measures (primary and secondary outcome data):
  - Feasibility data of the implant procedure.
  - Biosafety data of the implant procedure.
  - Effects of the implant on patient's growth, development and life quality.
  - Adverse events

The data collection process will be performed in a validated electronic Case Report Form (eCRF), by capturing data from participants enrolled in the study. Each participant's demographics, medical history, treatment details, and outcome measures will be entered in real time by trained site staff using an intuitive eCRF interface. Automated validation checks immediately flag any discrepancies, ensuring data accuracy from the outset. Regular source data verification will be conducted by the CRA to confirm that the data recorded aligns with the original documentation, maintaining compliance with regulatory standards.

DOCUMENTATION AND METADATA

As an advanced therapies clinical trial, the data will be accompanied by all the protocols and documents associated to this trial. The trial was approved by the Spanish Medicines Agency.

ETHICS AND LEGAL COMPLIANCE

As a clinical trial approved by the Spanish Medicines Agency, all legal issues are covered. The project has been approved by several ethics and research committees, including:

- Committee of Ethics in Research with Medicinal Products (CEIm) in Seville, reference FIB-BIO-2023-03 (date of approval, November 21th, 2023).
- Authorized by the Spanish Medicines Agency (*Agencia Española de Medicamentos y Productos Sanitarios, AEMPS*), reference 2023-506913-23-00/ID:10008 (date of approval, November 21st, 2023).

STORAGE AND BACKUP

As an advanced therapies clinical trial, the sponsor and the PIs will securely custody the data for at least twenty-five years following the completion of the trial.

Data will be preserved in digital form and custodied at the University Hospital Virgen de las Nieves, following all the security measures established for this type of studies, controlled by the clinical trial monitorization committee.

The PIs and the monitor are the only persons authorized to access the data. Other researchers could request partial access to the data.

DATA SHARING STATEMENT

Individual deidentified participant data will be shared, including data dictionaries, to promote transparency and further research in the field. The data shared will include demographic information, clinical outcomes and any adverse events reported during the trial. In addition to participant data, the following documents will be available: protocol and informed consent.

The results of the clinical trial will be made publicly available in the Clinical Trials Information System (CTIS) and on ClinicalTrials.gov. Since the trial is being conducted in Spain, it will adhere to the European regulations for the publication of results, which require that results be published within 12 months of trial completion. Additionally, the main

results and conclusions will be published in open-access journals and spread in scientific meetings and congresses to further disseminate our findings to the scientific community.

Non-personal data and data that are not considered to have ethical concerns or sensitive character will be deposited, whenever possible, in public repositories such as Zenodo or Digibug. Restrictions will affect the personal data and other data that could be sensitive or have ethical concerns.

RESPONSIBILITIES AND RESOURCES

Data management will be performed by the PIs of the project and the professionals in the Andalusian Network for the design and translation of Advanced Therapies participating in the clinical trial.

The data will be delivered using public repositories such as Zenodo or Digibug.
