# Supplementary material for: Phase I-IIa clinical trial to evaluate the safety, feasibility and efficacy of the use of a palate mucosa generated by tissue engineering for the treatment of children with cleft palate: the BIOCLEFT study protocol
Source: BMJ Open. 2024 Dec 5;14(12):e093491. doi: 10.1136/bmjopen-2024-093491 (PMC11624797; doi:10.1136/bmjopen-2024-093491)
Supplement: online supplemental file 4 [file bmjopen-14-12-s004.pdf]

INFORMATION SHEET FOR THE PARTICIPATION OF THE SUBJECT IN THE CLINICAL TRIAL AND INFORMED CONSENT BY REPRESENTATION

(THIS IS AN ENGLISH TRANSLATION OF THE ORIGINAL DOCUMENT WRITTEN IN SPANISH.

THE DOCUMENT IN SPANISH IS AVAILABLE AT THE END OF THIS DOCUMENT)

Title: A phase I-IIa unicenter, controlled, open and randomized clinical trial to evaluate the safety, feasibility, and preliminary efficacy of a human fibrin-agarose, nanostructured, palate mucosa substitute generated by tissue engineering for the treatment of children with cleft palate.

Protocol code: FIB-BIO-2023-03

Eu CT number: 2023-506913-23-00

Promoter: Fundación para la Investigación Biosanitaria de Andalucía Oriental - Alejandro Otero (FIBAO)

Principal Investigator: Ricardo Fernández Valadés

Center: Hospital Universitario Virgen de las Nieves

BACKGROUND

This document aims to inform you about a clinical trial, which is a research study to find better treatments for children with Cleft Lip and Palate, as it is the case of your child, and we invite you to participate.

The study has been approved by the Research Ethics Committee of Seville, which belongs to the Network of Ethics Committees of the Public Health System of Andalusia, and by the Spanish Medicines Agency, in accordance with current regulations regarding clinical trials with medicines, the European Regulation 536/2014, of April 16 and the Royal Decree 1090/2015, of December 4.

Our intention is that you receive the correct and sufficient information so that you can decide whether or not you want your child to participate in this trial. To do this, read this information sheet carefully and ask your doctor any questions you may have. You are also given a copy of this document so that you can evaluate it and consult it with the people you consider appropriate.

VOLUNTARY PARTICIPATION

We invite you to participate in the study because your child has a palate defect (total unilateral cleft lip and palate).

Your child's participation is voluntary, so you can decide not to participate. If you decide to give your consent and participate in the study, you may change your decision at any time, without altering your relationship with your doctor or causing any harm to the health care provided to your child.

WHAT DOES THE CLINICAL TRIAL CONSIST OF?

Cleft lip and palate are defects found in the upper lip, the roof of the palate, or both. They occur when these facial structures do not close completely during pregnancy and development. Cleft lip and palate entail functional and aesthetic alterations of various structures that, depending on the case, produce alterations in the pronunciation of words, swallowing, deformity of the lips, nose and palate, the musculature of the pharynx and the position of the teeth, as well as possible alterations in the emergence of temporary or permanent teeth, and the growth of facial bones

In order to repair these defects, quite complex interventions are performed to improve the aesthetic appearance and function of the facial structures and, depending on the case, to improve feeding, by preventing food from escaping through the nose, to improve pronunciation, and to favor the correct alignment of both dental arches. The complexity of the defect can lead to the operated area not to have a completely normal external appearance.

The artificial palate, called BIOCLEFT, is an advanced therapy medicinal product. It is made from cells donated by your own child and cultured, and natural materials: fibrin (a protein obtained from blood), and agarose (a natural product extracted from algae). By processing the previously donated sample, cell cultures of the two main types of cells that make up the palate will be obtained in the laboratory. Once these cells are expanded in the laboratory, they will be introduced into a fibrin-agarose matrix to generate the human artificial palate.

GOALS OF THE STUDY

The objective of the present study is to confirm that it is possible and safe to use a BIOCLEFT oral mucosa substitute generated from a sample of your child's own mucosa, and whether this use improves the aesthetic and functional results of current surgical treatment in relation to the closure of the cleft areas, and a more correct reconstruction in children affected by cleft lip and palate, as it is the case of your son/daughter.

STUDY DESCRIPTION

This is a clinical trial carried out in a single hospital.

Patients will be selected from children treated at the Cleft Lip and Palate and Craniofacial Malformations Care Unit (CLPU) of the Virgen de las Nieves University Hospital in Granada, and will be included in the study whenever they meet all the inclusion criteria, but not the exclusion criteria.

15 children affected by complete unilateral cleft lip and palate will participate in this study. All of them will undergo standard reconstruction surgery.

Of the 15 children, 10 patients will be treated with the standard reconstruction surgery, followed by covering the operated area with the novel product studied in this trial (experimental group), during the same surgical procedure, whereas the other 5 patients will receive only the standard reconstruction surgery.

The study will be carried out in 2 stages: the first 5 children to be included will all receive the study treatment. In a second stage, the remaining 10 children will have a 50% probability of receiving the investigational treatment and a 50% probability of receiving the standard treatment. Whether your child is assigned to the experimental group or to the control group, will depend on chance. You will be informed about the group assigned to your child.

Both surgical procedures will be performed under general anesthesia.

Children in both groups will receive the same usual reconstruction therapy and will undergo the same wound care, monitoring and evaluation protocol

An Independent Safety and Data Monitoring Committee has been established in the trial to analyze the results. Once the first 5 children have been treated, this Committee will decide, according to the data obtained, whether or not to continue with the inclusion of the remaining 10 patients.

The results of all your blood tests, as well as other analytical results, will be provided to the sponsor. These results are coded in such a way that the promoter will not know who owns these results. Positive results for HIV and viral hepatitis will be communicated to local health authorities as established by current regulations.

FOR HOW LONG SHALL I PARTICPATE IN THE STUDY?

Once you agree with the inclusion of your child in the study, your participation will last approximately 2 years. During this period, you will have to attend 12 visits: two visits before the standard surgical intervention and BIOCLEFT implant -if applicable-, one visit for the implant and 9 follow-up visits after the intervention.

The procedures that will be followed at each visit, as well as the number of visits, will be the same for all patients. The visit schedule is included in APPENDIX 1 to this document.

In addition to the visits described in the protocol, intermediate visits may be carried out if your doctor considers that these are needed.

RISKS AND DISCOMFORT ARISING FROM YOUR PARTICIPATION IN THE STUDY

Your participation in the study could be associated to several risks and discomforts that may arise from:

Participation in the Clinical Trial, since you and your child must attend all the visits listed in the protocol and in ANNEX 1, until the follow-up is completed.

Additionally, you will need to complete a series of clinical evaluations, and you will be asked to complete some questionnaires. In addition, in some of them, photographs of your child's face and molds of the dental arch will be taken, to check the evolution. Finally, the blood analyses that will be performed at the beginning of the study carries a minimal risk, and may cause discomfort such as pain and slight hematoma (accumulation of blood in the skin) in the puncture area.

To assess the aesthetic appearance and the changes that occur in response to the treatment, photographs of your child's head (face and skull) will be taken at different visits (Visit 1, 3, 10 and 12). These photographs

will be studied by an expert surgeon and may later be used in scientific publications. In the photograph, the eye area with the black spot will be hidden to avoid facial recognition of the patient.

The risks derived from surgery are common to the control group and the experimental group. Despite the correct surgical technique, undesirable effects derived from the intervention may occur:

- Frequent non-serious risks depend on the degree of alteration present in the lip, and the condition of the patient, and include: Hemorrhage or infection of the surgical wound, healing problems, separation of the edges of the wound or appearance of fistulas between the mouth and nose, asymmetry of the nose and lip contour, difficulty in pronouncing some sounds and finally, impossibility of complete closure.
- The most serious and rare risks that may occur include: Necrosis of any of the areas of the skin that is used to close the fissure of the palate, lip or nose. Although these complications may occur, complications derived from the use of operating materials (electric scalpel, thermal blanket, etc.) or postural complications are very rare.
- Other risks related to the anesthesia used: The anesthetic risk depends on many factors such as the type and severity of the pathology that motivates the operation, whether it is performed urgently, other diseases suffered by the patient, or his/her condition or age. All these parameters may increase side effects. The most common include: nausea and vomiting, throat conditions in case of intubation; vertigo and vision disorders, tremors, headache, itching, muscle and joint pain and back pain. In the hours following anesthesia, temporary disorders of memory, attention or behavior of the child may appear. The most serious are: aspiration of stomach contents into the lungs, although this is very rare if preoperative fasting has been respected, unforeseeable complications that could endanger your child's life such as a true allergic reaction, malignant hyperthermia (fever), respiratory failure or cardiac arrest. Death during anesthesia is very rare and is almost always the consequence of a set of simultaneous complications.

Risks derived from the investigational medical product:

To date, no toxic effects have been described due to the administration of the elements used to generate the artificial mucosa, including fibrin, agarose and oral mucosa cells. This does not exclude the possibility that some problems may occur. As it is an investigational treatment, this is one of the aspects to be assessed from a safety point of view.

The medication may contain traces of Gentamicin and Amphotericin B derived from the manufacturing process. If you know that your child is allergic to these medications, you should inform the medical personnel in charge of the intervention.

All necessary tests and treatments will be carried out so that the risks of the intervention and evaluation tests are reduced to a minimum.

WHAT ARE THE EXPECTED BENEFITS?

Based on current scientific evidence, it is expected that the proposed treatment will improve the repair of the clefted tissues and, as a consequence, your child may have an appearance similar to that of any other child who was not born with the injury, as well as the improvement of certain functions such as speech, swallowing, hearing, etc.

In any case, with your participation in the study you will contribute to improving knowledge about possible alternatives to treat this disease, thus collaborating in the scientific advancement of society.

WHAT IS MY COMMITMENT?

You agree to attend the visits with your child and undergo the scheduled tests, to notify any adverse event that happens to your child, whether or not it is related to the clinical trial, and to communicate any changes made to your child’s medication, if any. If you stop attending follow-up visits without having expressly revoked your consent, the sponsor may complete your clinical follow-up by accessing your medical history at the center and collecting data for the study.

Likewise, you must inform the different health professionals with whom you have a relationship that your child is participating in this clinical trial, warning them that, for safety reasons and possible contraindications with the investigational product. Current medication should not be modified without consulting the study doctor in the event that your child has any prescribed medication.

IF I HAVE ANY QUESTIONS OR QUESTIONS, WHO CAN I CONTACT?

You can **ask any questions you want** and learn more about this clinical trial, **now or at any time** during the course of the trial. Additionally, if you notice your child experiencing any adverse events, you should contact the study doctor immediately.

Study researcher: Dr. Ricardo Fernández Valadés.

Phone: ……………………………….

E-mail: ………………………………………………………………………………………

Hospital: Hospital Universitario Virgen de las Nieves

If you need emergency medical attention, you can go to your child's usual center. You must inform the different health professionals with whom you have a relationship that you are participating in this clinical trial and provide all possible them with information related to the study.

WHAT ALTERNATIVE TREATMENTS ARE THERE?

It is important that you know that if you do not want your child to participate in the proposed clinical trial, the researcher responsible for the development of the clinical trial will indicate the palliative or curative alternatives currently available for the management of the disease.

Do not hesitate to consult with the researcher responsible for the development of the clinical trial if you have any questions or if you need any clarifications on this issue.

EXPENSES AND ECONOMIC COMPENSATION

The Promoter of the study is in charge of the economic aspects of the study. To carry out the study, the Public Foundation FIBAO has signed the corresponding contract for its implementation. This is a clinical trial carried out within the public system. Neither the center nor the research team will receive any financial compensation. Your participation in the study will not entail any additional expense to the usual clinical practice of your disease.

WHAT TREATMENT WILL I RECEIVE WHEN MY PARTICIPATION IN THE STUDY WILL FINISH?

Once your participation in the study ends, your child will receive the treatment that his/her doctor considers most appropriate for his/her clinical situation.

INSURANCE POLICY

The Promoter of the study has an insurance policy that complies with current legislation (Royal Decree 1090/2015), which will provide compensation in the event of impairment of the health of your child or injuries that may occur related to his/her participation in the study, as long as these events are not a consequence of the disease itself or its evolution as a consequence of the ineffectiveness of the treatment. This policy has been contracted with the insurance company HDI Global. For more information regarding this section, please consult the principal investigator of the study at your center.

WHO HAS ACCESS TO MY DATA?

Both the promoter and the center will ensure that the principles contemplated in the data protection regulations, both national and European, are complied with.

For more information on confidentiality and protection of personal data, see APPENDIX 1.

WHAT IS MY DATA USED FOR?

Your data is necessary for the sponsor to develop the medicinal product, to obtain permission to introduce and keep it on the market, monitor its safety, and cover it with health insurance, that is, throughout the drug development program. Therefore, they will be used as planned in this study, as well as within the related research activities necessary for this drug development program in order to:

- understand how the study product and similar drugs work in the body (that is, evaluate the mode of action of the study product),
- better understand the studied disease and associated health problems,

- develop diagnostic tests for the disease,
- learn from this study to plan new studies or improve scientific analysis methods,
- publish research results in scientific journals or use them for educational purposes.

OTHER RELEVANT INFORMATION

A description of this clinical trial will be available at **http://reec.aemps.es as** required by Spanish legislation. You will also find information about the study at **https:// https://clinicaltrials.gov**. In addition, one year after the end of the study, a summary of its results (regardless of what they are) will be available in the European Union database, as well as a summary written in a language understandable to a person.

Any new information regarding the therapies used in the study that may affect your willingness for your child to participate in the study, which is discovered during your participation, will be communicated to you by your doctor as soon as possible.

If the new information implies any modification to the information sheet, you will be provided with the new version of the document, giving you the opportunity to decide if you want your child to continue participating in the study

WHEN WILL MY PARTICIPATION IN THE TRIAL END?

The investigator, the Sponsor, the Ethics Committee and the regulatory authorities that supervise this clinical trial may decide to withdraw your child from the study if they consider that it will be for your best interest.

- Once the **follow-up period has concluded**, which will last approximately 24 months, from signing the consent.
- **At any time if you decide**, you must notify the researcher.
- If the **study doctor decides to suspend your child's participation** in the trial, because a new medication with greater proven effectiveness has been approved, because he/she considers it to be the best for your child, or if you do not follow the procedures of the study clinical trial, you will receive an adequate explanation of the reason that caused your child to withdraw from the study.
- For **other unforeseen circumstances**, whenever the Promoter or the Health Authorities consider it appropriate.

In any case, you must follow the instructions given to you by the study doctor for an orderly completion of the trial.

WHAT WILL HAPPEN TO MY SAMPLES?

The blood samples collected will be associated with a code that can only be related to your child's identity and medical history by the study doctor/collaborators. The data derived from the use of these samples will be treated in the same way as the rest of the data obtained during the test. Your samples and associated data may be analyzed in various laboratories, for the same purposes of the study described, but always maintaining the confidentiality of your identity in accordance with current legislation.

ACKNOWLEDGEMENT

Whatever your decision is, the sponsor and research team would like to thank you for your time and attention.

PARTICIPANT CONSENT SHEET BEFORE WITNESSES AND/OR RELATIVES OR RELATIVES

**Title of the study:** A phase I-IIa unicenter, controlled, open and randomized clinical trial to evaluate the safety, feasibility, and preliminary efficacy of a human fibrin-agarose, nanostructured, palate mucosa substitute generated by tissue engineering for the treatment of children with cleft palate
**Protocol code**: FIB-BIO-2023-03 **Eu CT number:** 2023-506913-23-00

I,____*<<name of the parent or legal guardian>>*, as

☐ Father/Mother / ☐ Legal guardian,

I declare that I received a satisfactory explanation on the study procedure, its objectives, risks, benefits and alternatives, from Dr.:

____ *<<name of the researcher>>*, and I have read the study information sheet that has been provided to me, and:

☐ I had the opportunity to make any questions on the study

☐ I have received enough information on the study

☐ I have been speaking with

____ *<<name of the researcher>>*

☐ I understand that my participation is voluntary

☐ I understand that I can withdraw from the study:

- Whenever I wish,
- Without the need of giving any explanations
- Without any consequences of the care and treatment provided to my son/daughter

The participant accepts taking any biological simples for the objectives of the study:

☐ YES ☐ NO

The parents or legal guardian of the participant in the study wishes to be informed about any information derived from the study that may be relevant to his/her health:

☐ YES ☐ NO

I will receive a signed and dated copy of this informed consent document.

I freely give my consent for my child to participate in the study.

Signature of one of the parents or legal guardians:<br>Date:<br><br>Name and original written signature	Signature of the research and ID number:<br>Date:<br><br>Name and original written signature

Signature of another of the parent or legal guardian:<br>Date:<br><br>Name and original written signature	Signature of the research and ID number:<br>Date:<br><br>Name and original written signature

APPENDIX 1. Personal data protection related to the Patient Information Sheet and Informed Consent documents of the study

Title of the study:	A phase I-IIa unicenter, controlled, open and randomized clinical trial to evaluate the safety, feasibility, and preliminary efficacy of a human fibrin-agarose, nanostructured, palate mucosa substitute generated by tissue engineering for the treatment of children with cleft palate.
Code of the study:	FIB-BIO-2023-03
EU CT Number:	2023-506913-23-00
Promoter:	Fundación para la Investigación Biosanitaria de Andalucía Oriental-Alejandro Otero (FIBAO)
Principal Investigator:	Ricardo Fernández Valadés
Center:	Hospital Universitario Virgen de las Nieves

In accordance with the provisions of Regulation (EU) 2016/679 of the European Parliament and the European Council of April 27, 2016 on Data Protection and the Organic Law 3/2018, of December 5, on the Protection of Personal Data and guarantee of digital rights, it is important that you know the following information:

The Foundation for Biosanitary Research of Eastern Andalusia - Alejandro Otero (FIBAO) is the promoter of this study. It is based in Granada. Both the Center and the Promoter are responsible for their respective treatments, with each of them corresponding to the obligations derived from their activity. The center is responsible for all the data that appear in the medical record that can identify you, whilst the promoter is responsible for data that are collected in this study in a coded way. During your participation in the study, you will be identified by a code and neither the researcher nor the hospital will transfer to the sponsor any information that could directly identify you.

The list that relates the identification code and the data that identifies you (name, surname, medical record number...) is kept confidentially at your health center.

WHAT HAPPENS WITH CONFIDENTIALITY?

Access to your identified personal information will be restricted to the study doctor and collaborators, health authorities (Spanish Medicines Agency, foreign health authorities), the Ethics Committee for Research with Medicines (CEIm) and personnel authorized by the promoter (study monitors or auditors), when it is needed to verify the data, study procedures, and compliance with standards of good clinical practice, but always maintaining confidentiality. Your child's identity may be revealed in exceptional cases, such as medical emergencies for his/her health or legal requirements. Processing, communication and transfer of personal data of all participants will comply with the provisions of the applicable regulations.

Likewise, in the event of an adverse reaction, the identifying data may be communicated by the Center to the competent health authorities and to the insurance companies with which insurance has been contracted, in order to carry out the necessary steps.

The encrypted data may be transmitted to third parties and other countries, but in no case will it contain information that can directly identify your child, such as name and surname, initials, address, social security number, etc. In the event that this transfer occurs, it will be for the same purposes of the study described or for use in scientific publications, but always maintaining their confidentiality in accordance with current legislation.

FOR HOW LONG WILL MY DATA BE KEPT?

The Researcher and the Sponsor are obliged to retain the data collected for the study for at least 25 years after its completion. Afterwards, your personal information will only be maintained by your child's health care facility.

WHAT RIGHTS DO I HAVE?

With respect to the data, your child has the following rights that you may exercise before the main researcher and/or center:

- You can ask at any time what data are stored (right of access), who is using these data and for what purpose; you may request a copy of the personal data for your own use.
- You can request to receive a copy of the personal data provided by you to transmit these data to other people (portability).
- You can correct your child's personal data provided by you and limit the use of data that are incorrect (right of rectification and deletion).
- You can object to the use of personal data or restrict this use (right to object).

We remind you that there are some limitations in order to ensure the validity of the research and comply with the legal duties of the sponsor and drug authorization requirements. If you decide that your child stops participating in the trial or withdraws your consent to data processing, the data collected up to that point cannot be deleted. You should know that if you decide to withdraw consent regarding data processing, it could determine your termination of participation in the trial.

WHO CAN I CONTACT?

To exercise your rights, contact the principal investigator of the study (whose details appear on the information sheet above) or the Data Protection Officer of your Center, or the promoter.

Data Protection Delegate for all centers of Junta de Andalucía:

Email: dpd.sspa@juntadeandalucia.es

Data Protection Delegate for the promoter:

Email: dpd.csalud@juntadeandalucia.es

Likewise, you have the right to contact the Data Protection Agency if you are not satisfied (through the website *www.aepd.es*).

HOW WILL THE RESULTS BE COMMUNICATED?

A description of this clinical trial will be available at https://reec.aemps.es, as required by Spanish legislation.

The sponsor is obliged to publish the results, both positive and negative, of authorized clinical trials, preferably, in scientific journals before being disclosed to the non-specialized public, regardless of the obligations to publish the report of the results in the Spanish Registry of clinical studies (REec) and the provisions established in this regard in Regulation (EU) No. 536/2014 of the European Parliament and the European Council, of April 16, 2014.

When studies and research work on medicines are made public, aimed at the scientific community, the funds obtained by the author, for its development, and the source of financing, will be stated.

The anonymity of the subjects participating in the trial will be maintained at all times.

SAFEGUARD FOR THE PROTECTION OF YOUR PERSONAL DATA

Appropriate protective measures will be taken to protect encrypted data during and after the test, including:

- Access to encrypted data will be limited to persons subject to confidentiality obligations (including the obligation not to attempt to re-identify patients or decode clinical data).
- Encrypted data will be protected with security measures to prevent alteration, loss and unauthorized access and additional measures may be applied to prevent identification.
- Encrypted data will not be shared for direct marketing purposes or for other purposes that are not legal obligations or are not considered scientific research in accordance with current data protection legislation. In particular, they will not be used to make decisions about future services that may be offered to you, such as insurance.

ANNEX 1. CALENDAR OF VISITS AND PROCEDURES

Visit	Procedures and evaluations
**V1 Selection.** 4 months before the surgical procedure	☐ Informed consent signature<br>☐ Obtaining blood samples and photographs; Quality of Life Questionnaire.
**V2 (by phone).** 1 month before the surgical procedure	☐ To confirm that you can be included in the study, and that a group can be assigned to you.
**V3.** Surgical procedure	☐ Surgical procedure<br>☐ Evaluation by the ENT doctor, obtaining molds and photographs.
**V4, 5 & 6.** 1, 2 and 7 days after the surgical procedure	☐ Wound evaluation, including photographs
**V7.** 14 days after the surgical procedure	☐ Wound evaluation, Quality of Life questionnaire and photographs
**V8 & V9.** 1.5 and 4 months after the surgical procedure	☐ Wound evaluation and evaluation by the ENT doctor, if necessary.
**V10.** 9 months after the surgical procedure	☐ Evaluation, palate impressions and generation of molds and photographs. Evaluation by the ENT doctor if necessary. Quality of life questionnaire
**V11.** 15 months after the surgical procedure	☐ Wound evaluation. Language assessment by the speech therapist. Obtaining photographs
**V12. 2**4 months after the surgical procedure	☐ Wound evaluation. Quality of life questionnaire, photographs. Evaluation by the ENT doctor if necessary, and language assessment by the speech therapist.

HOJA DE INFORMACIÓN PARA LA PARTICIPACIÓN DEL SUJETO EN EL ENSAYO CLÍNICO Y CONSENTIMIENTO INFORMADO POR REPRESENTACIÓN

Título: Ensayo clínico fase I-IIa, aleatorizado, controlado, no enmascarado y unicéntrico, para evaluar la seguridad, factibilidad e indicios de eficacia del uso de un sustituto autólogo de mucosa palatina humana de fibrina-agarosa nanoestructurada generado por ingeniería tisular, en el tratamiento de pacientes con fisura palatina.

Código protocolo: FIB-BIO-2023-03

Eu CT number: 2023-506913-23-00

Promotor: Fundación para la Investigación Biosanitaria de Andalucía Oriental-Alejandro Otero (FIBAO)

Investigador Principal: Ricardo Fernández Valadés

Centro: Hospital Universitario Virgen de las Nieves

INTRODUCCIÓN

Nos dirigimos a usted para informarle sobre un ensayo clínico, que es un estudio de investigación para poder encontrar mejores tratamientos para niños con Fisura Labio Palatina, como la que presenta su hijo, en el que le invitamos a participar.

El estudio ha sido **aprobado** por el Comité de Ética de la Investigación con medicamentos de Sevilla, que pertenece a la Red de Comités de Ética del Sistema Sanitario Público de Andalucía, y por la Agencia Española de Medicamentos y Productos Sanitarios, de conformidad con la legislación vigente en materia de ensayos clínicos con medicamentos, el Reglamento Europeo 536/2014, de 16 de abril y el Real Decreto 1090/2015, de 4 de diciembre.

Nuestra intención es que usted reciba la información correcta y suficiente para que pueda decidir **si quiere o no que su hijo/a participe** en este ensayo. Para ello, lea esta hoja de información con atención y pregunte a su médico las dudas que le puedan surgir. Se le entrega, además, una copia de este documento para que pueda valorarlo y consultarlo con las personas que considere oportuno.

PARTICIPACIÓN VOLUNTARIA

Le invitamos a participar en el estudio porque su hijo/a presenta una lesión en el paladar consistente en una fisura unilateral total.

La **participación de su hijo/a es voluntaria**, por lo que puede decidir no participar. Si decide otorgar su consentimiento y que su hijo/a participe en el estudio, podrá cambiar su decisión en cualquier momento, sin que por ello se altere la relación con su médico, ni se produzca perjuicio alguno en su atención sanitaria.

¿EN QUÉ CONSISTE EL ENSAYO CLÍNICO?

Las fisuras del labio y del paladar son orificios o hendiduras del labio superior, del techo del paladar, o de ambos. Se producen cuando estas estructuras de la cara no se cierran por completo durante el embarazo. La fisura labio-palatina conlleva alteraciones funcionales y estéticas de varias estructuras que, según los casos, producen alteraciones de la pronunciación de las palabras, al tragar, deformidad de labios, nariz y paladar, de la musculatura de la faringe y de la posición de los dientes, así como posibles alteraciones en la salida de los dientes temporales o definitivos, y del crecimiento de los huesos de la cara.

Para cerrar estas fisuras se realizan intervenciones que son bastante complejas, y que están destinadas a mejorar el aspecto estético y la función, y según los casos la mejora de la alimentación, evitar la salida de comida por la nariz, mejora de la pronunciación, y el correcto alineamiento de ambas arcadas dentarias. La complejidad puede hacer que el aspecto exterior de la zona operada no sea totalmente normal.

Este paladar artificial, que llamamos BIOCLEFT, es un medicamento de terapia avanzada. Está elaborada a partir de células donadas por su propio hijo/a, en cultivo y materiales naturales: fibrina (proteína obtenida de sangre), y agarosa (producto natural extraído de algas). Mediante el procesamiento de la muestra donada previamente, se obtendrán en el laboratorio cultivos celulares de los dos tipos de células que conforman el paladar. Una vez multiplicadas estas células en el laboratorio serán introducidas en una matriz de fibrina y agarosa para fabricar el paladar artificial humano.

OBJETIVO DEL ESTUDIO

El **objetivo** del presente estudio es confirmar que es posible y seguro utilizar un sustituto de mucosa oral, BIOCLEFT, fabricado a partir de una muestra de la propia mucosa de su hijo/a, y si este uso mejora los resultados estéticos y funcionales del tratamiento quirúrgico habitual, en relación con el cierre de las zonas fisuradas, y una reconstrucción más correcta, en los niños/as que, como su hijo/a, presentan una fisura del labio y del paladar.

DESCRIPCIÓN DEL ESTUDIO

Se trata de un ensayo clínico que se realizará en un único hospital.

Los pacientes serán seleccionados de entre los que acudan a la Unidad de Cuidados de labio Leporino y paladar hendido y malformaciones craneofaciales (CLPU) del Hospital Universitario Virgen de las Nieves de Granada. y se incluirán en el mismo, siempre que cumplan todos los criterios de inclusión y ninguno de exclusión.

En este estudio participaran 15 niños, afectados de fisura labial y palatina completa unilateral. Todos ellos serán sometidos a la cirugía de reconstrucción habitual.

De los 15 niños, en 10 de ellos, en la misma intervención de la cirugía de reconstrucción habitual, la zona operada se recubrirá con el medicamento que se está estudiando (grupo experimental) y los otros 5 recibirán solamente la cirugía de reconstrucción habitual.

El estudio se realizará en 2 etapas: los 5 primeros niños que se incluyan, recibirán todos ellos el tratamiento en estudio. En una segunda etapa, los 10 niños restantes, tendrá una probabilidad del 50% de recibir el tratamiento en investigación y una probabilidad del 50% de recibir el tratamiento estándar. El que a su hijo/a le corresponda el grupo experimental o el grupo control, dependerá del azar. Usted sabrá en qué grupo le ha tocado participar.

Ambos procedimientos quirúrgicos se realizarán bajo anestesia general.

Los niños de ambos grupos recibirán la misma terapia de reconstrucción habitual y serán sometidos a un mismo protocolo de cuidado de la herida, seguimiento y evaluación.

En el ensayo se ha establecido un Comité Independiente de Seguridad y Monitorización de Datos para analizar los resultados que se vayan produciendo, y este Comité, una vez tratados los 5 primeros niños, según los datos obtenidos, decidirá si se continua o no con la inclusión de los 10 pacientes restantes.

Se facilitarán al promotor los resultados de todos sus análisis de sangre, así como otros resultados analíticos. Estos resultados están codificados de manera que el promotor no sabe a quién pertenecen dichos resultados. Los resultados positivos del VIH y de la hepatitis viral se comunicarán a las autoridades sanitarias locales tal como marca la legislación sanitaria.

¿CUÁNTO DURARÁ MI PARTICIPACIÓN EN EL ESTUDIO?

Una vez que usted acepte que su hijo/a participe en el estudio, su participación durará aproximadamente 2 años. Durante este periodo deberá acudir a 12 visitas: dos visitas antes de la intervención quirúrgica estándar e implante de BIOCLEFT, si le corresponde, una visita para el implante y 9 visitas de seguimiento tras la intervención.

Los procedimientos a seguir en cada visita, así como el número de visitas serán los mismos para todos los pacientes. El calendario de las visitas se recoge en el ANEXO 1 a este documento.

Además, de las visitas descritas en el protocolo, se podrán realizar todas las visitas intermedias que el médico responsable considere oportuno.

RIESGOS Y MOLESTIAS DERIVADOS DE SU PARTICIPACIÓN EN EL ESTUDIO

Su participación en el estudio podría ocasionar un conjunto de riesgos e incomodidades que se pueden derivar de:

<u>La participación en el Ensayo Clínico</u>, ya que usted y su hijo/a deberán atender a todas las visitas recogidas en el protocolo y en el ANEXO 1, hasta completar el seguimiento.

Además, será necesario realizar una serie de evaluaciones clínicas y se le pedirá que complete algunos cuestionarios. Además, en algunas de ellas se tomarán fotografías de la cara y moldes de la arcada dentaria de su hijo/a, para ir comprobando la evolución. Por último, la extracción de sangre que se realizará al inicio del estudio conlleva un riesgo mínimo, pudiendo producir molestias como dolor y hematoma leve (acúmulo de sangre en la piel) en la zona de punción.

Para valorar la apariencia estética y los cambios que se vayan produciendo en respuesta al tratamiento, se le realizarán a su hijo/a, fotografías de la cabeza (cara y cráneo), en diferentes visitas (Visita 1, 3, 10 y 12). Estas fotografías serán estudiadas por un cirujano experto, y podrán ser empleadas posteriormente en publicaciones científicas. En la fotografía se ocultará la zona de los ojos con mancha negra para evitar el reconocimiento facial del paciente.

<u>Los riesgos derivados de la cirugía</u> son comunes al grupo control y al grupo experimental. A pesar de la correcta técnica quirúrgica pueden presentarse efectos indeseables derivados de la intervención:

- <u>Los riesgos frecuentes y no graves dependen del grado de alteración presente en el labio, y del estado del paciente,</u> son: Hemorragia o infección de la herida quirúrgica, problemas de cicatrización, separación de los bordes de la herida o

aparición de fístulas entre la boca y la nariz, asimetría de la nariz y del contorno del labio, dificultad para la pronunciación de algunos sonidos y por último imposibilidad de cierre completo.

- Los riesgos más graves e infrecuentes que pueden darse son: Necrosis de alguna de las zonas de la piel que se utiliza para cerrar la fisura del paladar, del labio o de la nariz; pueden ocurrir, pero son muy infrecuentes las complicaciones derivadas del uso de material de quirófano (bisturí eléctrico, manta térmica, etc.) o las complicaciones posturales.
- Otros riesgos son los relacionados con la anestesia empleada: El riesgo anestésico depende de muchos factores como son el tipo y la gravedad de la patología que motiva la operación, si se realiza de urgencia, otras enfermedades que padezca el/la paciente, o su edad, puede aumentar los efectos secundarios. Los más frecuentes son: náuseas y vómitos, afecciones en la garganta en caso de intubación; vértigos y trastornos de la visión, temblores, dolor de cabeza, picores, dolores musculares, articulares y dolor de espalda. En las horas que siguen a la anestesia pueden aparecer trastornos pasajeros de memoria, de la atención o del comportamiento del niño. Los más graves son: paso del contenido del estómago a los pulmones, aunque es muy raro si se ha respetado el ayuno preoperatorio, complicaciones imprevisibles que puedan poner en peligro la vida de su hijo como una reacción alérgica verdadera, una hipertermia (fiebre) maligna, insuficiencia respiratoria o una parada cardiaca. La muerte en el curso de una anestesia es muy rara y casi siempre es la consecuencia de un conjunto de complicaciones simultáneas.

Riesgos derivados de medicamento en investigación:

Hasta el momento no se han descrito efectos tóxicos por la administración de los elementos usados para la fabricación de la mucosa artificial, como son la fibrina, la agarosa y las células de la mucosa oral. Esto no significa que no pudiera producirse alguno. Al tratarse de un tratamiento en investigación, éste es uno de los aspectos a valorar desde el punto de vista de seguridad.

El medicamento puede contener trazas derivadas del proceso de fabricación de Gentamicina y de Anfotericina B. Si usted conoce que su hijo presente alergia a estos medicamentos, debe ponerlo en conocimiento del personal facultativo encargado de la intervención.

Se harán todas las pruebas y tratamientos necesarios para que los riesgos de la intervención y de las pruebas de evaluación se reduzcan al mínimo.

¿CUÁLES SON LOS BENEFICIOS ESPERADOS?

Basándonos en la evidencia científica, se espera que el tratamiento propuesto mejore el cierre de las zonas fisuradas y como consecuencia que su hijo/a presente un aspecto parecido al de cualquier otro niño que no haya nacido con la lesión, así como la mejora de ciertas funciones como son el habla, la deglución, audición, etc.

En cualquier caso, con su participación en el estudio contribuirá a mejorar el conocimiento sobre posibles alternativas para tratar su enfermedad, colaborando de este modo en el avance científico de la sociedad.

¿CUÁL ES MI COMPROMISO?

Usted se compromete a **acudir junto con su hijo/a a las visitas y a que se someta a las pruebas programadas,** notificar cualquier **evento adverso** que le suceda a su hijo/a, tenga o no relación con el ensayo clínico y a comunicar los **cambios realizados en su medicación,** si los hay. En caso de que deje de acudir a las visitas de seguimiento sin haber revocado expresamente su consentimiento, el promotor podrá completar su seguimiento clínico mediante el acceso a su historial médico en el centro y recogida de datos para el estudio.

Asimismo, deberá **comunicar a los distintos profesionales de la salud** con los que tenga relación que su hijo/a está participando en este ensayo clínico, advirtiéndoles que, por motivos de seguridad y posibles contraindicaciones con el medicamento en investigación, no deben modificar la medicación que esté tomando sin consultar antes con el médico del estudio en el caso de que tenga medicación prescrita.

SI TENGO ALGUNA DUDA O CONSULTA, ¿A QUIÉN ME DIRIJO?

Puede **realizar todas las preguntas que desee** y saber más sobre este ensayo clínico, **ahora o en cualquier momento** en el curso del mismo. Además, si observa que su hijo/a experimenta cualquier **evento adverso**, debe contactar inmediatamente con el médico del estudio.

Investigador del estudio: D. Ricardo Fernández Valadés.

Teléfono: ……………………………….

Email: ………………………………………………………………………………………

Hospital: Hospital Universitario Virgen de las Nieves

En caso de necesitar atención médica de urgencia puede dirigirse al centro habitual de su hijo/a, deberá **comunicar a los distintos profesionales de la salud** con los que tenga relación que está participando en este ensayo clínico y facilitar toda la información posible relativa al estudio.

¿QUÉ TRATAMIENTOS ALTERNATIVOS EXISTEN?

Es importante que sepa que en caso de no querer que su hijo/a participe en el ensayo clínico que se le propone, el investigador responsable del desarrollo del ensayo clínico le indicará las alternativas paliativas o curativas actualmente disponibles para el manejo de su enfermedad.

No dude en consultar con el investigador responsable del desarrollo del ensayo clínico cualquier duda que tenga o si necesita cualquier aclaración sobre esta cuestión.

GASTOS Y COMPENSACIÓN ECONÓMICA

El Promotor del estudio gestiona los aspectos económicos del mismo. Para la realización del estudio la Fundación Pública Andaluza para la Investigación Biosanitaria en Andalucía Oriental- Alejandro Otero ha firmado el correspondiente contrato para su realización. Se trata de un ensayo clínico realizado dentro del sistema público. Ni el centro ni el equipo investigador recibirán compensación económica alguna. Su participación en el estudio no le supondrá ningún gasto adicional a la práctica clínica habitual de su enfermedad.

¿QUÉ TRATAMIENTO RECIBIRÉ CUANDO FINALICE MI PARTICIPACIÓN EN EL ESTUDIO?

Una vez finalice su participación en el estudio, su hijo/a recibirá el tratamiento que su médico considere más adecuado para su situación clínica.

PÓLIZA DE SEGURO

El Promotor del estudio dispone de una póliza de seguros que se ajusta a la legislación vigente (Real Decreto 1090/2015), que le proporcionará la compensación e indemnización en caso de menoscabo de la salud de su hjo/a o de lesiones que pudieran producirse en relación con su participación en el estudio, siempre que no sean consecuencia de la propia enfermedad que se estudia o de la evolución propia de la misma como consecuencia de la ineficacia del tratamiento. Dicha póliza, ha sido contrata con la compañía aseguradora **HDI Global**. Si desea más información relativa a este apartado, consulte con el investigador principal del estudio en su centro.

¿QUIÉN TIENE ACCESO A MIS DATOS?

Tanto el promotor como el centro se asegurarán de que se cumplan los principios contemplados en la normativa de protección de datos, tanto nacional como europea

Para obtener más información sobre la confidencialidad y la protección de datos de carácter personal, consulte el Apéndice 1.

¿PARA QUÉ SE UTILIZAN MIS DATOS?

Sus datos son necesarios para que el promotor desarrolle el medicamento, obtenga permiso para introducirlo y mantenerlo en el mercado, supervise su seguridad y lo cubra el seguro de salud, es decir, durante todo el programa de desarrollo de medicamentos. Por lo tanto, se utilizarán según lo planeado en este estudio, así como dentro de las actividades de investigación relacionadas necesarias para este programa de desarrollo de medicamentos con el fin de:

- comprender cómo funcionan el medicamento del estudio y medicamentos similares en el organismo (es decir, evaluar el modo de acción del medicamento del estudio),
- comprender mejor la enfermedad estudiada y los problemas de salud asociados,
- desarrollar pruebas de diagnóstico para la enfermedad,
- aprender del presente estudio para planificar nuevos estudios o mejorar los métodos de análisis científico,
- publicar los resultados de la investigación en revistas científicas o utilizarlos con fines educativos.

OTRA INFORMACIÓN RELEVANTE

Una descripción de este ensayo clínico estará disponible en **http://reec.aemps.es** según exige la legislación española. Encontrará igualmente información sobre el estudio en **https:// https://clinicaltrials.gov**. Además, transcurrido un año desde la finalización del estudio, estarán disponibles en la base de datos de la Unión Europea el resumen de sus resultados (independientemente de cuáles sean), así como un resumen redactado en un lenguaje comprensible para una persona.

Cualquier nueva información referente a las terapias utilizadas en el estudio y que pueda afectar a su disposición para que su hijo/a participe en el estudio, que se descubra durante su participación, le será comunicada por su médico lo antes posible.

Si la nueva información implica alguna modificación de la hoja de información, se le facilitará la nueva versión del documento, dándosele la oportunidad de decidir si desea que su hijo/a continúe su participación en el estudio.

¿CUÁNDO FINALIZA MI PARTICIPACIÓN EL ENSAYO?

El investigador, el Promotor, el Comité de ética y las autoridades reguladoras que supervisan este ensayo clínico pueden decidir retirar a su hijo/a si consideran que es lo mejor para él/ella.

- Una vez **concluido el periodo de seguimiento** que tendrá una duración de aproximadamente 24 meses, desde la firma del consentimiento.
- **En cualquier momento si usted lo decide**, debiendo comunicarlo al investigador.
- Si el **médico del estudio decidiera suspender la participación de su hijo/a** en el ensayo, por haberse aprobado un nuevo medicamento con mayor eficacia demostrada, por considerar que es lo mejor para su hijo/a, o si usted no siguiese los procedimientos del ensayo clínico, usted recibirá una explicación adecuada del motivo que ha ocasionado la retirada de su hijo/a del estudio.
- Por otras **circunstancias imprevistas,** siempre que el Promotor o las Autoridades Sanitarias lo consideren oportuno.

En todo caso, deberá seguir las indicaciones que le transmita el médico del estudio, para una finalización ordenada del ensayo.

¿QUÉ OCURRIRÁ CON MIS MUESTRAS?

Las muestras de sangre recogidas se asociarán a un código que solo podrá ser relacionado con la identidad de su hijo/a y con su historia clínica por el médico del estudio/colaboradores. Los datos que se deriven de la utilización de estas muestras se tratarán del mismo modo que el resto de datos que se obtengan durante el ensayo. Sus muestras y los datos asociados podrán ser analizados en diversos laboratorios, para los mismos fines del estudio descrito, pero siempre manteniendo la confidencialidad de su identidad de acuerdo a la legislación vigente.

AGRADECIMIENTO:

Sea cual sea su decisión, el promotor y el equipo de investigación quieren agradecer su tiempo y atención.

HOJA DE CONSENTIMIENTO DE PARTICIPANTE ANTE TESTIGOS Y/O FAMILIAR/PERSONA VINCULADA DE HECHO

Título: Ensayo clínico Fase I-IIa, unicéntrico, controlado, abierto y aleatorizado, para evaluar la seguridad, factibilidad e indicios de eficacia del uso de un sustituto autólogo de mucosa palatina humana de fibrina-agarosa nanoestructurada generado por ingeniería tisular, en el tratamiento de pacientes con fisura palatina.
Código de protocolo: FIB-BIO-2023-03
Eu CT number: 2023-506913-23-00

Yo,____ ________________<<*nombre y apellidos* ***del padre o tutor legal*** >> como

☐ Padre/madre / ☐ Tutor legal, afirmo que he recibido una explicación satisfactoria sobre el procedimiento del estudio, su finalidad, riesgos, beneficios y alternativas por parte del D/Dª

____

<<*nombre y apellidos del investigador*>> y que he leído la hoja de información que se me ha entregado sobre el estudio, de modo que:

☐ Ha podido hacer preguntas sobre el estudio

☐ Ha recibido suficiente información sobre el estudio.

☐ Ha hablado con

____

<<*nombre del investigador*>>

☐ Comprende que su participación es voluntaria.

☐ Comprende que puede retirarse del estudio:

- Cuando quiera,
- Sin tener que dar explicaciones y
- Sin que esto repercuta en los cuidados médicos de mi hijo/a.

El participante acepta que se tomen muestras de su hijo/a para los mismos fines del estudio descrito:

☐SÍ ☐ NO

El padre, madre o tutor legal del participante desea que le comuniquen la información derivada de la investigación que pueda ser relevante para su salud:

☐SÍ ☐ NO

Recibiré una copia firmada y fechada de este documento de consentimiento informado.

Presto libremente mi conformidad para que mi hijo/a participe en el estudio.

Firma del representante legal, primer progenitor o persona vinculada de hecho:<br>Fecha:<br><br>Nombre, firma y fecha de puño y letra por el firmante	Firma del investigador y Nº de colegiado<br>Fecha:

Firma del segundo progenitor (o tutor legal) del menor:<br><br><<Nombre y fecha de puño y letra del firmante>>	Fecha:<br><br><<fecha de puño y letra del firmante>>

Apéndice 1. Protección de datos de carácter personal relativo al documento Hoja de información al paciente y CI del estudio

Título del estudio	Ensayo clínico Fase I-IIa, unicéntrico, controlado, no enmascarado y aleatorizado, para evaluar la seguridad, factibilidad e indicios de eficacia del uso de un sustituto autólogo de mucosa palatina humana de fibrina-agarosa nanoestructurada generado por ingeniería tisular, en tratamiento de pacientes con fisura palatina.
Código del estudio	FIB-BIO-2023-03
EU CT Number	2023-506913-23-00
Promotor	Fundación para la Investigación Biosanitaria de Andalucía Oriental-Alejandro Otero (FIBAO)
Investigador Principal	Ricardo Fernández valades
Centro	Hospital Universitario Virgen de las Nieves

De conformidad con lo establecido en el Reglamento (UE) 2016/679 del Parlamento europeo y del Consejo de 27 de abril de 2016 de Protección de Datos y en la Ley Orgánica 3/2018, de 5 de diciembre, de Protección de Datos Personales y garantía de los derechos digitales, es importante que conozca la siguiente información:

La Fundación para la Investigación Biosanitaria de Andalucía Oriental-Alejandro Otero (FIBAO) es el promotor de este estudio. Tiene su sede en Granada. Tanto el Centro como el Promotor son responsables de sus respectivos tratamientos, correspondiendo a cada uno de ellos las obligaciones derivadas de su actividad. El centro es el responsable de todos los datos que figuren en la historia y que puedan identificarle y el promotor de los que se recogen en este estudio de forma codificada; es decir, durante su participación en el estudio se le identificará mediante un código y ni el investigador, ni el hospital transferirán al promotor información alguna que pueda identificarle directamente.

La lista que relaciona el código de identificación con los datos que le identifican (nombre, apellido, número de historia clínica…) se guardan de manera confidencial en su centro sanitario.

¿QUÉ OCURRE CON LA CONFIDENCIALIDAD?

El acceso a su información personal identificada quedará restringido al médico del estudio y colaboradores, autoridades sanitarias (Agencia Española de Medicamentos y Productos Sanitarios, autoridades sanitarias extranjeras), al Comité de Ética de la Investigación con medicamentos (CEIm) y personal autorizado por el promotor (monitores del estudio o auditores), cuando lo precisen para comprobar los datos, procedimientos del estudio, y el cumplimiento de normas de buena práctica clínica; pero siempre manteniendo la confidencialidad de los mismos. La identidad de su hijo/a podría ser revelada en casos excepcionales, como situaciones de urgencia médica para su salud o requerimiento legal. El tratamiento, la comunicación y la cesión de los datos de carácter personal de todos los participantes se ajustarán a lo dispuesto en la normativa aplicable.

Igualmente, en caso de producirse una reacción adversa, los datos identificativos podrán ser comunicados por el Centro a las autoridades sanitarias competentes y a las compañías aseguradoras con las que se hubiese contratado un seguro, a fin de llevar a cabo las gestiones que resulten necesarias.

Los datos codificados pueden ser transmitidos a terceros y a otros países, pero en ningún caso contendrán información que pueda identificar directamente a su hijo/a, como nombre y apellidos, iniciales, dirección, nº de la seguridad social, etc. En el caso de que se produzca esta cesión, será para los mismos fines del estudio descrito o para su uso en publicaciones científicas, pero siempre manteniendo la confidencialidad de los mismos de acuerdo a la legislación vigente.

¿DURANTE CUÁNTO TIEMPO SE GUARDARÁN MIS DATOS?

El Investigador y el Promotor están obligados a conservar los datos recogidos para el estudio al menos hasta 25 años tras su finalización. Posteriormente, su información personal solo se conservará por el centro para el cuidado de la salud de su hijo/a.

¿QUÉ DERECHOS TENGO?

Con respecto a los datos, su hijo/a tiene los siguientes derechos que usted podrá ejercer ante el investigador principal y/o centro:

- Puede preguntar en cualquier momento qué datos se están guardando (derecho de acceso), quién los usa y con qué fin; puede solicitar una copia de los datos personales para su propio uso.

- Puede solicitar recibir una copia de los datos personales proporcionados por usted para transmitirlos a otras personas (portabilidad).
- Puede corregir los datos personales de su hijo/a proporcionados por usted y limitar el uso de datos que sean incorrectos (derecho de rectificación y supresión).
- Puede oponerse al uso de los datos personales o restringirlo (derecho de oposición)

Le recordamos que existen algunas limitaciones con objeto de garantizar la validez de la investigación y cumplir con los deberes legales del promotor y los requisitos de autorización de medicamentos. Si decide que su hijo/a deje de participar en el ensayo o retirar su consentimiento sobre el tratamiento de los datos no se podrán eliminar aquellos datos recogidos hasta ese momento. Debe saber que si decide retirar el consentimiento sobre el tratamiento de los datos podría determinar su cese en la participación en el ensayo.

¿CON QUIÉN CONTACTO?

Para ejercitar sus derechos, diríjase al investigador principal del estudio (cuyos datos aparece en la página 8 de la hoja de información superior) o al Delegado/a de Protección de Datos de su Centro, o del promotor.

Delegado de Protección de Datos para todos los centros de la Junta de Andalucía:

Email: dpd.sspa@juntadeandalucia.es

Delegado de Protección de Datos del promotor:

Email: dpd.csalud@juntadeandalucia.es

Así mismo, tiene derecho a dirigirse a la Agencia de Protección de Datos si no quedara satisfecho (a través de su página web *www.aepd.es*).

¿CÓMO SE COMUNICARÁN LOS RESULTADOS?

Una descripción de este ensayo clínico estará disponible en https://reec.aemps.es, según exige la legislación española.

El promotor está obligado a publicar los resultados, tanto positivos como negativos, de los ensayos clínicos autorizados, preferentemente, en revistas científicas antes de ser divulgados al público no sanitario, con independencia de las obligaciones de publicación del informe de los resultados en el Registro Español de estudios clínicos

(REec) y de lo establecido al respecto en el Reglamento (UE) n.º 536/2014 del Parlamento Europeo y del Consejo, de 16 de abril de 2014.

Cuando se hagan públicos estudios y trabajos de investigación sobre medicamentos, dirigidos a la comunidad científica, se harán constar los fondos obtenidos por el autor, por o para su realización, y la fuente de financiación.

Se mantendrá en todo momento el anonimato de los sujetos participantes en el ensayo.

SALVAGUARDA PARA LA PROTECCIÓN DE SUS DATOS PERSONALES

Se adoptarán las medidas de protección apropiadas para proteger los datos codificados durante y después del ensayo, entre ellas:

- El acceso a los datos codificados quedará limitado a personas sujetas a obligaciones de confidencialidad (incluida la obligación de no intentar volver a identificar a los pacientes ni descodificar los datos clínicos).
- Los datos codificados se protegerán con medidas de seguridad para evitar su alteración, pérdida y accesos no autorizados y podrán aplicarse medidas adicionales que eviten la identificación.
- Los datos codificados no se compartirán con fines de comercialización directa ni para otros fines que no sean obligaciones legales o que no se consideren investigación científica de conformidad con la legislación vigente en materia de protección de datos. En particular, no se utilizarán para tomar decisiones sobre futuros servicios que se le pudieran ofrecer, como un seguro.

ANEXO 1. CALENDARIO DE VISITAS Y PROCEDIMIENTOS

Visita	Procedimientos/Evaluaciones
**V1 Selección.** 4 meses antes de la cirugía.	• Firma del consentimiento informado<br>• Obtención de muestras de sangre, fotografías y Cuestionario de Calidad de Vida.
**V2 (Telefónica).** 1 mes antes de la cirugía.	• Para confirmar que puede ser incluido en el estudio, y poder asignarle un grupo.
**V3 (cirugía)**	• Intervención quirúrgica<br>• Revisión por el otorrino, obtención de moldes y fotografías.
**V4, 5 y 6** (A las 24, 48 horas, y 7 días de la cirugía	• Revisión de la herida, incluyendo fotografías
**V7.** A los 14 días de la cirugía	• Revisión de la herida, cuestionario de Calidad de vida y fotografías
**V8 y V9.** Al mes y medio y 4 meses y medio de la cirugía	• Revisión de la herida y revisión por el otorrino si es necesario.
**V10.** A los 9 meses de la cirugía	• Revisión, impresiones del paladar y generación de moldes y fotografías. Revisión por el otorrino si es necesario. Cuestionario de Calidad de vida.
**V11.** A los 15 meses de la cirugía	• Revisión de la herida. Valoración del lenguaje por el logopeda y fotografías
**V12.** A los 24 meses de la cirugía	• Revisión de la herida. Cuestionario Calidad de vida, fotografías, valoración de la audición por el otorrino si es necesario y del lenguaje por el logopeda.
