# Supplementary material for: Phase I-IIa clinical trial to evaluate the safety, feasibility and efficacy of the use of a palate mucosa generated by tissue engineering for the treatment of children with cleft palate: the BIOCLEFT study protocol
Source: BMJ Open. 2024 Dec 5;14(12):e093491. doi: 10.1136/bmjopen-2024-093491 (PMC11624797; doi:10.1136/bmjopen-2024-093491)
Supplement: Supplementary file 5 [file bmjopen-2024-093491.reviewer_comments.pdf]

PEER REVIEW HISTORY

BMJ Open publishes all reviews undertaken for accepted manuscripts. Reviewers are asked to complete a checklist review form and are provided with free text boxes to elaborate on their assessment. These free text comments are reproduced below.

ARTICLE DETAILS

Title (Provisional)

A phase I-IIa clinical trial to evaluate the safety, feasibility, and efficacy of the use of a palate mucosa generated by tissue engineering for the treatment of children with cleft palate: the BIOCLEFT study protocol

Authors

España-López, Antonio; Fernández-Valadés, Ricardo; Cubiles, Elisa; Garzón, Ingrid; Martin-Piedra, Miguel Angel; Carriel, Víctor; Campos, Fernando; Martínez-Plaza, Adoración; Vallejo, Daniel; Liceras-Liceras, Esther; Chato-Astrain, Jesús; García-García, Oscar Dario; Sánchez-Porras, David; Ávila-Fernández, Paula; Etayo-Escanilla, Miguel; Quijano, Blanca; Aguilar, Elisabet; Campos, Antonio; Carmona, Gloria; ALAMINOS, MIGUEL

VERSION 1 - REVIEW

**Reviewer**	**1**
**Name**	**Cornefjord, Måns**
**Affiliation**	**Skåne University Hospital, Department of Plastic and Reconstructive Surgery**
**Date**	**11-Oct-2024**
**COI**	

Comments to the Authors

Thank you for conducting this interesting and novel research in the field of cleft palate. I found the described study very ambitious, and I hope that the results will allow you to continue with the later stages of clinical testing of this product. However, I have some remarks and suggested changes/clarifications to the study protocol:

- In general, there are a few spelling and grammar mistakes that need correction. There are also a few places where the choice of words makes the text a bit difficult to understand. I would consider sending the manuscript for language

review before resubmission. Also, please ensure that patient-first language is used throughout (for example on page 4 row 6).

- There methodology is well-described overall. However, I would suggest a few clarifications:
    - What is meant by the "safety period" of 30 days between patients (page 6 rows 21-22)? Why was a period of 30 days chosen?
    - Some of the exclusion criteria listed on pages 6-7 are "unnecessary" as they are covered by the inclusion criteria, for example children whose guardians *do* or *do not* accept the inclusion of their child. Consider removing exclusion criteria that are covered by the inclusion criteria.
    - How were the children that donated oral mucosa at the time of cheiloplasty (one of the inclusion criteria) selected?
    - The description of the surgical procedure in the intervention group (page 7 rows 39-40) gives the impression that the palatal mucosa substitute will be applied after the palate repair (as described on page 7 rows 30-38) is complete. I would believe that it is going to be applied during the palate repair, before closure, as it should cover the denudated palatine bone? Please consider clarifying this. Also, will it be applied to the lateral von Langenbeck incisions as well, or only in the midline?
    - Figure 2 is very illustrative, but some of the information in the figure could be added to the text on page 8 as well. This includes for example at which time points the follow-up visits will take place.
    - What is meant by "cranio-maxillo-facial images" (page 8 rows 21-22), and how will they be used? How will speech be evaluated at visit 11 (page 8 rows 38-39).
    - Could the questionnaire used to assess feasibility of the procedure be described in further detail or even attached as a supplement (page 8 rows 50-53)?
    - In general, I believe that the secondary outcomes such as effects on surgical site healing, speech, cranio-maxillo-facial growth, and aesthetics will be very difficult to assess with such a short follow-up time, something that is also mentioned by the authors.
    - Perhaps, a description of how the results will be presented could be included in the Method section.
- The discussion describes the rationale behind the study, the limitations, and potential further research in an adequate way.
- The SPIRIT checklist is attached and completed. The inclusion and exclusion criteria listed (page 20 rows 17-36) are not entirely the same as in the study protocol. Please consider using the same criteria.

My recommendation would be minor revisions as described above for this study protocol.

Reviewer 2

Name Refahee, Shaimaa Mohsen

Affiliation Oral and Maxillofacial Surgery Department, Faculty of Dentistry, Fayoum University

Date 21-Oct-2024

COI

1]abstract section

- there was no result section

- please remove the ethical approval part from the abstract section

2] introduction section

-lines 7-11, please remove this phrases, not needed

- line 12, ofc(the stences not began with abbreviations)

-line 28: there were different techniques used for surgical repair of cleft palate as furlow with buccinator flap have minimal effect on the maxillary growth (Mamdouh Ahmed Aboulhassan1 et al 2023, Effects of two flap palatoplasty versus furlow palatoplasty with buccal

myomucosal flap on maxillary arch dimensions in patients with cleft

palate at the primary dentition stage: a cohort study) please add this reference

- line 40, not begain with abbreviation

3] methods section

- lines 17,18:gold standard is the surgical repaire not left untreated, it is not ethicaly accepted

-line 23 , please define of uranostaphylorraphy

4] results

- there is no results section

5] outcome analysis

-please clarify TAPQOL and how you assessed the esthetic outcome by aesthetic appearence scale

thank you

VERSION 1 - AUTHOR RESPONSE

Reviewer: 1

Dr. Måns Cornefjord, Skåne University Hospital, Lund University

Comments to the Author:

Comments to the Authors

Thank you for conducting this interesting and novel research in the field of cleft palate. I found the described study very ambitious, and I hope that the results will allow you to continue with the later stages of clinical testing of this product.

Authors' response (AU): Thank you very much for your kind comments, which have significantly contributed to improve the quality of the manuscript.

However, I have some remarks and suggested changes/clarifications to the study protocol:

• In general, there are a few spelling and grammar mistakes that need correction. There are also a few places where the choice of words makes the text a bit difficult to understand. I would consider sending the manuscript for language review before resubmission. Also, please ensure that patient-first language is used throughout (for example on page 4 row 6).

AU: The manuscript has been thoroughly revised and corrected by a professional native editor, and the patient-first language has been considered, as suggested by the reviewer.

• There methodology is well-described overall. However, I would suggest a few clarifications:

o What is meant by the "safety period" of 30 days between patients (page 6 rows 21-22)? Why was a period of 30 days chosen?

AU: The main objective of this early-phase clinical trial is to determine if the novel advanced therapies medicinal product grafted in patients is safe for the patients. As this is the first time that this type of product is implanted in humans, we established a minimum follow-up time of 30 days between patient and patient with the objective of evaluating the short-term side effects of the implant on each patient, before enrolling the next patient in the study. Please, note that this safety period was a requirement of the Spanish Medicines Agency in a different clinical trial that we previously carried out in patients with severe cornea damage that were grafted with a bioengineered cornea substitute (González-Andrades et al., 2017).

This relevant information hs been included in the revised manuscript (Page 4, Lines 20-24).

González-Andrades, M., et al. (2017). A study protocol for a multicentre randomised clinical trial evaluating the safety and feasibility of a bioengineered human allogeneic nanostructured anterior cornea in patients with advanced corneal trophic ulcers refractory to conventional treatment. BMJ Open 7, e016487. doi: 10.1136/bmjopen-2017-016487

o Some of the exclusion criteria listed on pages 6-7 are "unnecessary" as they are covered by the inclusion criteria, for example children whose guardians do or do not accept the inclusion of their child. Consider removing exclusion criteria that are covered by the inclusion criteria.

AU: We agree with the reviewer. However, please, note that one of the considerations of the Editor to this version of the manuscript was the need of describing the inclusion and exclusion criteria in the same way that these criteria were included in the trial registers. As we already registered the clinical trial including this redundant information, we must now include the same information in the manuscript. In addition, the last comment of the reviewer also considers the need of describing these criteria as done in the study protocol previously registered.

o How were the children that donated oral mucosa at the time of cheiloplasty (one of the inclusion criteria) selected?

AU: All patients with non-syndromic cleft palate with unilateral cleft lip are normally proposed to donate a small oral mucosa sample at the moment of the lip repair surgery (cheiloplasty). This sample corresponds to the remaining tissue that is usually discarded after the surgical procedure performed at the age of 3-6 months. This biopsy is kept and custodied at the GMP facility where the bioartificial palate mucosa will be then generated. If the legal guardians of the patient accept the child to participate in the present clinical trial (at the age of 10-14 months), this biopsy will be used for the generation of the bioartificial palate mucosa.

This relevant information has been included in the methods and Analysis section of the revised manuscript (Page 4, Lines 43-47).

o The description of the surgical procedure in the intervention group (page 7 rows 39-40) gives the impression that the palatal mucosa substitute will be applied after the palate repair (as described on page 7 rows 30-38) is complete. I would believe that it is going to be applied during the palate repair, before closure, as it should cover the denudated palatine

bone? Please consider clarifying this. Also, will it be applied to the lateral von Langenbeck incisions as well, or only in the midline?

AU: The bioartificial palate mucosa will be grafted after the von Langenbeck procedure has been performed, and this bioengineered tissue will be applied only to the lateral incisions where the palatine bone has been denudated. Please, note that the objective of the present study is to determine whether or not this artificial tissue could contribute to prevent the growth and development alterations found in children subjected to bone denudation during the von Langenbeck procedure.

This relevant information has been clarified in the revised manuscript (Page 5, Lines 43-44).

o Figure 2 is very illustrative, but some of the information in the figure could be added to the text on page 8 as well. This includes for example at which time points the follow-up visits will take place.

AU: This information has been included in the text, as requested by the reviewer (Page 6, Lines 21-22 and 39-43).

o What is meant by "cranio-maxillo-facial images" (page 8 rows 21-22), and how will they be used? How will speech be evaluated at visit 11 (page 8 rows 38-39).

AU: Cranio-maxillo-facial images correspond to photographs taken from the patient's head, face and mouth and palate. These images will be evaluated to determine the main parameters analyzed in this study.

Evaluation of the patient's speech will be carried out by an expert speech therapist using the conventional analysis methods used by these specialists for the clinical evaluation of patients with cleft palate. In short, the ability of the patient to articulate speech and the resonance patterns associated with this speech will be evaluated, including an analysis of hypernasality, resonance of specific vowels and consonants, detection of articulation errors (compensatory or not), etc. These characteristics of the patient speech will be collected and evaluated by the speech therapists, who will record a conversational speech sample for 2-3 minutes, which will be later analyzed. Then, single word articulation tests will be applied to assess specific sounds.

This information has been included in the revised manuscript (Page 6, Lines 26-27 and Page 7, Lines 39-46).

o Could the questionnaire used to assess feasibility of the procedure be described in further detail or even attached as a supplement (page 8 rows 50-53)?

AU: The questionnaire has been included as Supplementary Table S1 of the revised manuscript, as suggested (cited in Page 6, Line 2).

o In general, I believe that the secondary outcomes such as effects on surgical site healing, speech, cranio-maxillo-facial growth, and aesthetics will be very difficult to assess with such a short follow-up time, something that is also mentioned by the authors.

AU: We agree with the reviewer that an accurate evaluation of these parameters will require longer follow-up times. However, in this initial clinical trial, we will perform a preliminary initial analysis of these parameters, which will need to be evaluated in deep in a future study.

o Perhaps, a description of how the results will be presented could be included in the Method section.

AU: This is a very good suggestion. A specific section regarding the data sharing and results diffusion plan has been included at the end of the methods section of the manuscript (Page 7, Lines 49-52 and Page 8, Lines 1-2).

• The discussion describes the rationale behind the study, the limitations, and potential further research in an adequate way.

AU: Thank you very much for your kind comments.

• The SPIRIT checklist is attached and completed. The inclusion and exclusion criteria listed (page 20 rows 17-36) are not entirely the same as in the study protocol. Please consider using the same criteria.

AU: Criteria have been revised and rewritten to match those that are described in the study protocol published in Clinicaltrials.gov (Page 4, Lines 48-49 and Page 5, Lines 1-21).

My recommendation would be minor revisions as described above for this study protocol.

Reviewer: 2

Dr. Shaimaa Mohsen Refahee, Oral and Maxillofacial Surgery Department, Faculty of Dentistry, Fayoum University

Comments to the Author:

1]abstract section

- there was no result section

Authors' response (AU): Thank you very much for your kind comments, which have significantly contributed to improve the quality of the manuscript. Regarding the abstract, it is important to note that this study has not generated any results yet. Instead, it is a protocol for a clinical trial that is ongoing. Now, we have included a sentence in the abstract, and also in the methods section of the manuscript, stating that results will be published in relevant public databases as soon as these results are available (Page 2, Lines 21-22; Page 7, Lines 49-52 and Page 8, Lines 1-2).

- please remove the ethical approval part from the abstract section

AU: Please, note that this is not a research manuscript, but a protocol manuscript. In this regard, the authors guidelines of the journal BMJ Open, available at https://bmjopen.bmj.com/pages/authors#submission_guidelines, state that "Abstract: this should be structured with the following sections. Introduction; Methods and analysis; Ethics and dissemination".

2] introduction section

-lines 7-11, please remove this phrases, not needed

AU: The referred sentences have been removed from the manuscript text, as suggested by the reviewer (Page 3, Lines 1-2).

- line 12, ofc(the stences not began with abbreviations)

AU: This sentence has been modified, as requested (Page 3, Line 4).

-line 28: there were different techniques used for surgical repair of cleft palate as furlow with buccinator flap have minimal effect on the maxillary growth (Mamdouh Ahmed Aboulhassan1 et al 2023, Effects of two flap palatoplasty versus furlow palatoplasty with buccal myomucosal flap on maxillary arch dimensions in patients with cleft palate at the primary dentition stage: a cohort study) please add this reference

AU: This reference has been included in the introduction section, along with a sentence describing the main results reported by this article (Page 3, Lines 19-21).

- line 40, not begain with abbreviation

AU: This sentence has been modified, as requested (Page 3, Line 26).

3] methods section

- lines 17,18:gold standard is the surgical repaire not left untreated, it is not ethicaly accepted

AU: The gold-standard group used in our study is a group of patients subjected to uranostaphylorrhaphy surgical repair of the palatal defect. As the reviewer states, we do not have any untreated group of patients. This sentence has been rewritten for clarity (Page 4, Lines 5-8).

-line 23 , please define of uranostaphylorrhaphy

AU: This procedure has been described in the methods section of the revised manuscript (Page 3, Lines 10-18). Please note that this is the gold-standard treatment applied to all cleft palate children treated in our Unit.

4] results

- there is no results section

AU: As stated above, this is a protocol manuscript, and results are not available yet. However, a specific section regarding the data sharing and results diffusion plan has been included at the end of the methods section of the manuscript (Page 7, Lines 49-52 and Page 8, Lines 1-2).

5] outcome analysis

-please clarify TAPQOL and how you assessed the esthetic outcome by aesthetic appearence scale

AU: A description of both the TAPQOL and the esthetic results analysis has been included in the revised manuscript (Page 7, Lines 31-36).

thank you

Reviewer: 1 competing interests.: Not applicable.

Reviewer: 2 competing interests.: not applicable
